# Mitochondrial GCN5L1 regulates glutaminase acetylation and hepatocellular carcinoma

**DOI:** 10.1002/ctm2.852

**Published:** 2022-05-11

**Authors:** Taotao Zhang, Yunlong Cui, Yanjin Wu, Jiahui Meng, Linmeng Han, Jiaqi Zhang, Chunyu Zhang, Chenxi Yang, Lu Chen, Xue Bai, Kai Zhang, Kaiyuan Wu, Michael N. Sack, Lingdi Wang, Lu Zhu

**Affiliations:** ^1^ Department of Pharmacology Tianjin Key Laboratory of Inflammatory Biology The Province and Ministry Co‐sponsored Collaborative Innovation Center for Medical Epigenetics School of Basic Medical Sciences Tianjin Medical University Tianjin China; ^2^ Hepatobiliary Surgery Department Tianjin Medical University Cancer Institute and Hospital Tianjin China; ^3^ Department of Physiology and Pathophysiology Tianjin Key Laboratory of Cell Homeostasis and Major Diseases School of Basic Medical Sciences Tianjin Medical University Tianjin China; ^4^ Department of Biochemistry and Molecular Biology School of Basic Medical Sciences Tianjin Medical University Tianjin China; ^5^ Laboratory of Mitochondrial Biology and Metabolism NHLBI National Institutes of Health Bethesda Maryland USA

**Keywords:** GCN5L1, glutaminase, HCC, mitochondria acetylation, mTORC1

## Abstract

**Background:**

Glutaminolysis is a critical metabolic process that promotes cancer cell proliferation, including hepatocellular carcinoma (HCC). Delineating the molecular control of glutaminolysis could identify novel targets to ameliorate this oncogenic metabolic pathway. Here, we evaluated the role of general control of amino acid synthesis 5 like 1 (GCN5L1), a regulator of mitochondrial protein acetylation, in modulating the acetylation and activity of glutaminase to regulate HCC development.

**Methods:**

Cell proliferation was determined by MTT, 2D and soft agar clone formation assays and orthotopic tumour assays in nude mice. GLS1/2 acetylation and activities were measured in cells and tumours to analyse the correlation with GCN5L1 expression and mTORC1 activation.

**Results:**

Hepatic GCN5L1 ablation in mice markedly increased diethylnitrosamine (DEN)‐induced HCC, and conversely, the transduction of mitochondrial‐restricted GCN5L1 protected wild‐type mice against HCC progression in response to DEN and carbon tetrachloride (CCl_4_) exposure. GCN5L1–depleted HepG2 hepatocytes enhanced tumour growth in athymic nude mice. Mechanistically, GCN5L1 depletion promoted cell proliferation through mTORC1 activation. Interestingly, liver–enriched glutaminase 2 (GLS2) appears to play a greater role than ubiquitous and canonical tumour–enriched glutaminase 1 (GLS1) in promoting murine HCC. Concurrently, GCN5L1 promotes acetylation and inactivation of both isoforms and increases enzyme oligomerisation. In human HCC tumours compared to adjacent tissue, there were variable levels of mTORC1 activation, GCN5L1 levels and glutaminase activity. Interestingly, the levels of GCN5L1 inversely correlated with mTORC1 activity and glutaminase activity in these tumours.

**Conclusions:**

Our study identified that glutaminase activity, rather than GLS1 or GLS2 expression, is the key factor in HCC development that activates mTORC1 and promotes HCC. In the Kaplan–Meier analysis of liver cancer, we found that HCC patients with high GCN5L1 expression survived longer than those with low GCN5L1 expression. Collectively, GCN5L1 functions as a tumour regulator by modulating glutaminase acetylation and activity in the development of HCC.

## Graphical abstract

1. Mitochondrial GCN5L1 regulates proliferation and tumorigenesis in hepatocellular carcinoma (HCC) via glutamine metabolism.
2. Deletion of general control of amino acid synthesis 5 like 1 (GCN5L1) enhances both glutaminase 1 (GLS1) and glutaminase 2 (GLS2) activities by altering acetylation and oligomerisation.
3. Compound 968 inhibits GLS2 to suppress diethylnitrosamine (DEN)‐ and carbon tetrachloride (CCl_4_)‐induced HCC development.
4. Our study suggests that targeting glutaminase or mTORC1 activity could be a potential therapeutic treatment for HCC expressing lower levels of GCN5L1.

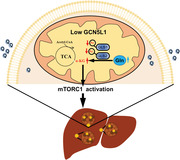

## 1 INTRODUCTION

Liver cancer, being aggressive, is the third leading cause of cancer‐related deaths worldwide, with a poor 5‐year survival rate.^1^ Hepatocellular carcinoma (HCC) is the major form of liver cancer, representing approximately 75% of all primary hepatic carcinomas.^2^ Cancer metabolism has attracted increasing attention in cancer research in recent decades since targeting metabolic alterations is emerging as a new strategy for cancer therapy.^3^, ^4^ During tumour initiation and invasion, cancer cells reprogramme their metabolic properties to meet energy demands to survive or adapt to the tumour environment. Increased aerobic glycolysis, fatty acid synthesis and glutaminolysis are common cancer metabolic signatures.^5^, ^6^, ^7^ Mitochondria function as biosynthetic organelles where metabolic intermediates contribute to nucleotide biosynthesis, macromolecule synthesis and redox control. It has been shown that mitochondria play a central role in metabolic adaptation to promote cellular growth and proliferation.^8^

Glutamine is the most abundant circulating amino acid and it is converted to α‐ketoglutarate to enter the tricarboxylic acid cycle (TCA) through glutaminase, glutamate dehydrogenase or aminotransferases to generate adenosine triphosphate (ATP) and glutathione (GSH) to maintain redox homeostasis. Glutaminase is the rate‐limiting enzyme in glutaminolysis and has two isoforms, GLS1 (known as GLS, or KGA) and GLS2. GLS1 is ubiquitously expressed in healthy tissues and many types of cancer,^9^ whereas GLS2 is primarily enriched in the liver. Emerging evidences have shown that HCC is addicted to glutamine.^10^, ^11^ However, with the development of HCC, the liver isoforms switch, with a relative decrease in GLS2 expression and increased GLS1 expression in comparison to the adjacent normal liver tissue.^12^ Enhanced GLS1 transcription is driven by c‐Myc, which is the primary oncogene in liver cancer.^13^ However, the contribution of GLS2 to HCC development and to cell proliferation remains unclear.^14^, ^15^ At the same time, protein acetylation has emerged as a prevalent posttranslational modification (PTM) in mitochondria to modulate enzymatic activity, and whether mitochondrial GLS acetylation is modulated to support hepatic tumour cell growth remains unknown.

Published research has shown the critical role of mTORC1 activation in HCC development and recurrence,^16^, ^17^, ^18^ and glutaminolysis underpins HCC through its metabolic consumption.^11^ Glutamine utilisation in HCC appears to be controlled by glutamine uptake and/or the glutamine metabolic rate.^11^, ^16^ C‐Myc is the most frequently overexpressed oncogene in HCC development and plays a role in mTORC1 activation via transcriptional induction of glutamine transporters.^16^ Emerging evidence has also linked mTORC1 activation to glutamine and lipid metabolism,^19^, ^20^ which indicates the critical role of mTORC1 in energy sensing and metabolic alteration to regulate cell growth. However, it remains unclear whether glutamine metabolism, which is controlled by glutaminase activity, contributes to mTORC1 activation in HCC.

The general control of amino acid synthesis 5 like 1 (GCN5L1) protein has been found to specifically regulate mitochondrial protein acetylation. It has also been shown that GCN5L1 and mitochondrial protein acetylation are dramatically decreased during carbon tetrachloride (CCl_4_)‐induced murine liver regeneration. Loss of GCN5L1 activates GLS2 activity by reducing its acetylation to promote glutaminolysis, followed by mTORC1 activation to support hepatocyte growth.^21^ GLS1 and GLS2 share sequence homology, especially in their catalytic domains. Thus, we hypothesised that GCN5L1 may regulate both GLS1 and GLS2 acetylation and activity to modulate glutaminolysis and thereby modulate the development of HCC. In the current study, we demonstrated that the mitochondrial protein acetylation regulator GCN5L1 functions as an HCC tumour suppressor. Loss of GCN5L1 significantly enhanced diethylnitrosamine (DEN)‐induced murine HCC, whereas overexpression of mitochondrial‐restricted GCN5L1 reversed murine tumour growth. Deletion of GCN5L1 in human HCC cell lines activated the mTORC1 pathway by enhancing glutaminolysis to promote cell proliferation, which appeared to be regulated by GCN5L1‐level‐dependent modulation of GLS1 and GLS2 acetylation and oligomerisation. The analysis of clinical samples showed that mTORC1 was partially activated in HCC specimens compared with adjacent tissues, although this activity did not correlate with the expression of c‐Myc or glutamine transporters or with GLS1/GLS2 protein levels. In contrast, mTORC1 activation was significantly correlated with enhanced glutaminase activity and decreased GCN5L1 expression in HCC, further supporting that GCN5L1 could modulate the mTORC1 pathway via the regulation of acetylation and GLS1 and GLS2 activity to control HCC development.

## 2 MATERIALS AND METHODS

### 2.1 Human tumour samples

HCC specimens and clinical information were obtained from the Hepatobiliary Surgery Department, Tianjin Medical University Cancer Institute and Hospital. This study was approved by the Ethics Committee of Tianjin Medical University Cancer Institute and Hospital (number: bc2021150).

### 2.2 Mouse studies

Animal protocols were approved by the Animal Care and Use Committee of Tianjin Medical University. Animals were maintained in an specific pathogen‐free environment with a 12‐hour light/dark cycle and housed with free access to water and a normal chow diet. BALB/c nude mice for orthotopic tumour xenografts were purchased from Beijing Vital River Laboratory Animal Technology Co., Ltd. GCN5L1 liver knockout (LKO) mice with C57BL/6J background have been described previously^22^ and compared to flox/flox littermates as controls.

The DEN‐induced HCC model was generated as previously described.^23^ Two‐week‐old GCN5L1 LKO mouse and the littermate controls were intraperitoneally injected with 25 mg/kg DEN. After 23 weeks of normal chow feeding, the mice were euthanized, and liver tissues were collected. The HCC mouse model combining DEN and CCl_4_ was described previously.^24^ Briefly, 2‐week‐old wild‐type (WT) mice were intraperitoneally administered DEN (25 mg/kg i.p.), followed by 10 or 20 injections every week of CCl_4_ (5 ml/kg i.p., 10% dissolved in olive oil) at 4 weeks of age. For in vivo hepatic mitochondrial GCN5L1 (MtG) expression, adeno‐associated virus (AAV)‐TBG‐MtG or EGFP was delivered into mice (1 × 10^11^ total genome copies) intravenously in 100 μl phosphate‐buffered saline (PBS) per mouse. For tumour evaluation and orthotopic mouse model establishment, the methods are described in detail in the Supporting Information.

### 2.3 Constructs and antibodies

Plasmids expressing human GLS1 and GLS2 were cloned into the pShuttle‐3xFlag, pShuttle‐Myc or pET28A vector. Mitochondrial‐restricted GCN5L1 (MtG) was previously described.^22^ The lentiviral sgGCN5L1, sgGLS1 or sgGLS2 vectors were established via ligation of synthesised oligos (Table S1) into the lentiCRISPR‐v1 vector. Rabbit anti‐GCN5L1 was a kind gift from Dr. Michael Sack (NHLBI, NIH, Bethesda, USA). Rabbit anti‐p‐p70S6K (9234), p70S6K (9202), ACK (9441), VDAC (4661), and tubulin (2146) were purchased from Cell Signaling Technology. Mouse anti‐flag (F1804) was purchased from Sigma. Rabbit anti‐c‐Myc (10828‐1‐AP) and GLS1 (12855‐1‐AP) were purchased from Proteintech. Rabbit anti GLS2 (AP6650D) was purchased from Abcepta and β‐actin (AC026) was purchased from ABclonal. HRP goat anti‐rabbit (AS014) or HRP anti‐mouse (AS003) were purchased from ABclonal.

### 2.4 Mitochondria isolation

Mitochondria were purified from cells or murine livers by a two‐step centrifuge strategy according to a previous publication.^21^ In brief, liver tissues or HepG2 cells were thoroughly homogenised with buffer for mitochondrial isolation (225 mM mannitol, 75 mM sucrose, 0.5% BSA, 0.5 mM EGTA and 30 mM Tris–HCl pH 7.4) and then centrifuged at 800 g and 4°C for 5 min. The supernatant was centrifuged at 9000 g and 4°C for 10 min. The pellet was collected as the mitochondrial fraction.

### 2.5 Cross‐linking analysis of glutaminase oligomerisation

A total of 5 μg of flag‐GLS1 or flag‐GLS2 plasmid was transfected into GCN5L1 KO or control HepG2 cells, and 36 h after transfection, flag‐GLS1 or flag‐GLS2 was purified by anti‐flag antibody and eluted by 3 × flag peptide (150 μg/ml). 10 μl of eluted sample was run on SDS gels as a loading control. 1 mM DSS (Sigma) was added to purified flag‐GLS1 or flag‐GLS2 for 30 min at RT. Cross‐linked samples were run on SDS gels and then anti‐flag antibody was used for immunoblotting analysis.

### 2.6 Glutaminase activity assay

Glutaminase activity assays were previously described.^21^ Briefly, mitochondria from HepG2 cells or HCC samples were used for endogenous glutaminase activity assays. To test the efficiency of glutaminase inhibitors, a plasmid encoding human GLS1 or GLS2 was transfected into 293T cells, and 2 h before cell harvest, inhibitors were incubated with the cells. Mitochondria were isolated and lysed in lysis buffer (50 mM HEPES pH 7.9, 100 mM NaCl and 0.05% Triton X‐100). Fifty μg of mitochondrial protein was incubated in 50 mM Tris‐acetate (pH 8.6), 0.25 mM ethylenediaminetetraacetic acid (EDTA) and 20 mM glutamine for a volume of 90 μl at 37°C for 1 h and was stopped by adding 10 μl of ice‐cold 2.4 M hydrogen chloride (HCl). The quenched reaction mixture was incubated with a reaction mixture containing 160 mM Tris‐HCl (pH 9.4), 400 mM hydrazine, 5 mM adenosine diphosphate (ADP), 2 mM nicotinamide adenine dinucleotide (NAD) and 7.5 U/ml glutamate dehydrogenase for a final volume of 220 μl. The reaction mixture was incubated at 37°C for 1 h, and the absorbance at 340 nm was recorded for each sample against a water blank before (A1) and after the final (A2) mixture incubation. The subtraction (A2‐A1), which indicates NADH generation, represented glutaminase activity for each sample. The residual protein samples were used for concentration analysis or immunoblotting against HA or Myc antibody as a loading control to calculate enzyme activity.

### 2.7 In vitro acetylation assay

GLS1 or GLS2 purified from BL21 cells with/without soluble mitochondrial proteins (100 μg) were incubated with different concentrations of acetyl‐CoA (0‐1.5 mM) in triethanolamine‐buffered saline (TBS) (50 mM Tris‐HCl, 150 mM NaCl, pH 8.0 at 37°C) with trichostatin A (TSA) and nicotinamide (NAM) at 37°C for 6 h, and then the samples were analysed by western blotting to detect protein acetylation.

### 2.8 Survival analysis

The effects of the GCN5L1 gene on the survival of patients with liver cancer were determined using the Kaplan–Meier plotter online survival analysis tool ( https://kmplot.com/analysis/ ). To obtain sufficient patient samples, the overall survival (OS), progression‐free survival (PFS) and disease‐free survival (DSS) modes were used to conduct all the analyses.

### 2.9 Statistical analysis

Data are expressed as the mean ± standard error of the mean (SEM) unless indicated. For statistical analysis, tests for normality and homogeneity were performed first, then tested for significance by one‐way or two‐way ANOVA, followed by post hoc tests indicated in the figure legends, or by unpaired two tailed *t* test. Statistical significances are denoted as N.S. (not significant; *P* > .05), *P < .05, **P < .01, ***P < .001.

Detailed methods are included in the Supporting Information.

## 3 RESULTS

### 3.1 Mitochondrial GCN5L1 functions as a tumour suppressor in murine HCC development

**FIGURE 1:**
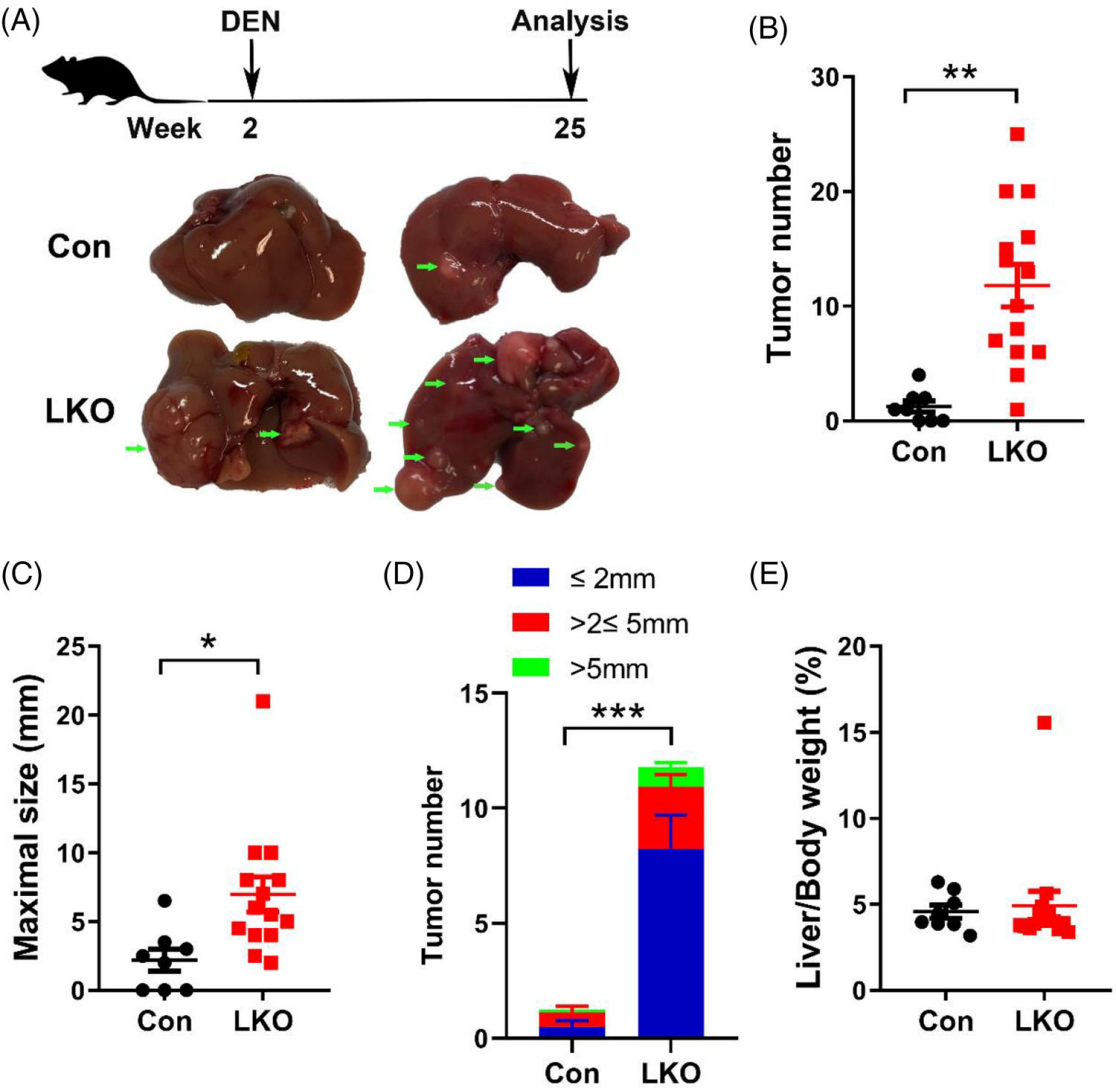
General control of amino acid synthesis 5 like 1 (GCN5L1) liver knockout mice (LKO) promotes diethylnitrosamine (DEN)‐induced hepatocellular carcinoma (HCC) development. Two‐week‐old male GCN5L1 LKO mice and their flox/flox littermates were i.p. injected with DEN (25 mg/kg body weight) and were maintained on normal chow (n = 8 for flox/flox, n = 14 for LKO). Mice were euthanized at 25 weeks of age for HCC analysis. (A) Schematic of the experimental design. Representative images of mouse livers from mice with the indicated genotypes. Arrows indicate tumours. (B) Tumour number. (C) Maximal size of tumours. (D) Number of liver tumours of the indicated sizes. (E) Ratio of liver weight to body weight. Values are expressed as the mean ± standard error of the mean, *P < .05, **P < .01 versus respective control groups by two‐tailed unpaired Student's *t* test or Bonferroni's multiple comparisons test

To first determine whether GCN5L1 depletion‐induced glutaminolysis predisposes to HCC development, we subjected GCN5L1^flox/flox^ (control) and GCN5L1^flox/flox^‐albumin‐Cre (GCN5L1 LKO) mice to a single dose of DEN at 2 weeks of postnatal life. Twenty‐three weeks post‐DEN injection, the GCN5L1 LKO mice showed a decreased body weight, which would be compatible with a large tumour burden. The experimental cohort was then euthanized at this time for subsequent analysis. This duration of exposure to DEN‐mediated tumour development is consistent with prior models where concurrent insults, including a high‐fat diet or CCl_4_ injection, promoted DEN‐induced HCC development.^23^, ^24^ Here, we found that DEN injection resulted in a low tumour burden (evidence of small lesions) in 62.5% of control mice (five out of eight mice) (Figure 1A). In contrast, GCN5L1 LKO mice had a higher tumour burden, and 100% (total 14 mice) of GCN5L1 LKO mice had liver tumours and displayed a profound enhancement of tumour number and maximal tumour size in comparison with control mice (Figure 1B,C). Further distribution assessment indicated that the number of various tumour sizes was significantly increased in LKO mice (Figure 1D). These results indicated that deletion of hepatic GCN5L1 promoted DEN‐induced HCC development. Despite this, we observed a similar liver/body weight ratio of GCN5L1 LKO to control mice (Figure 1E).

**FIGURE 2:**
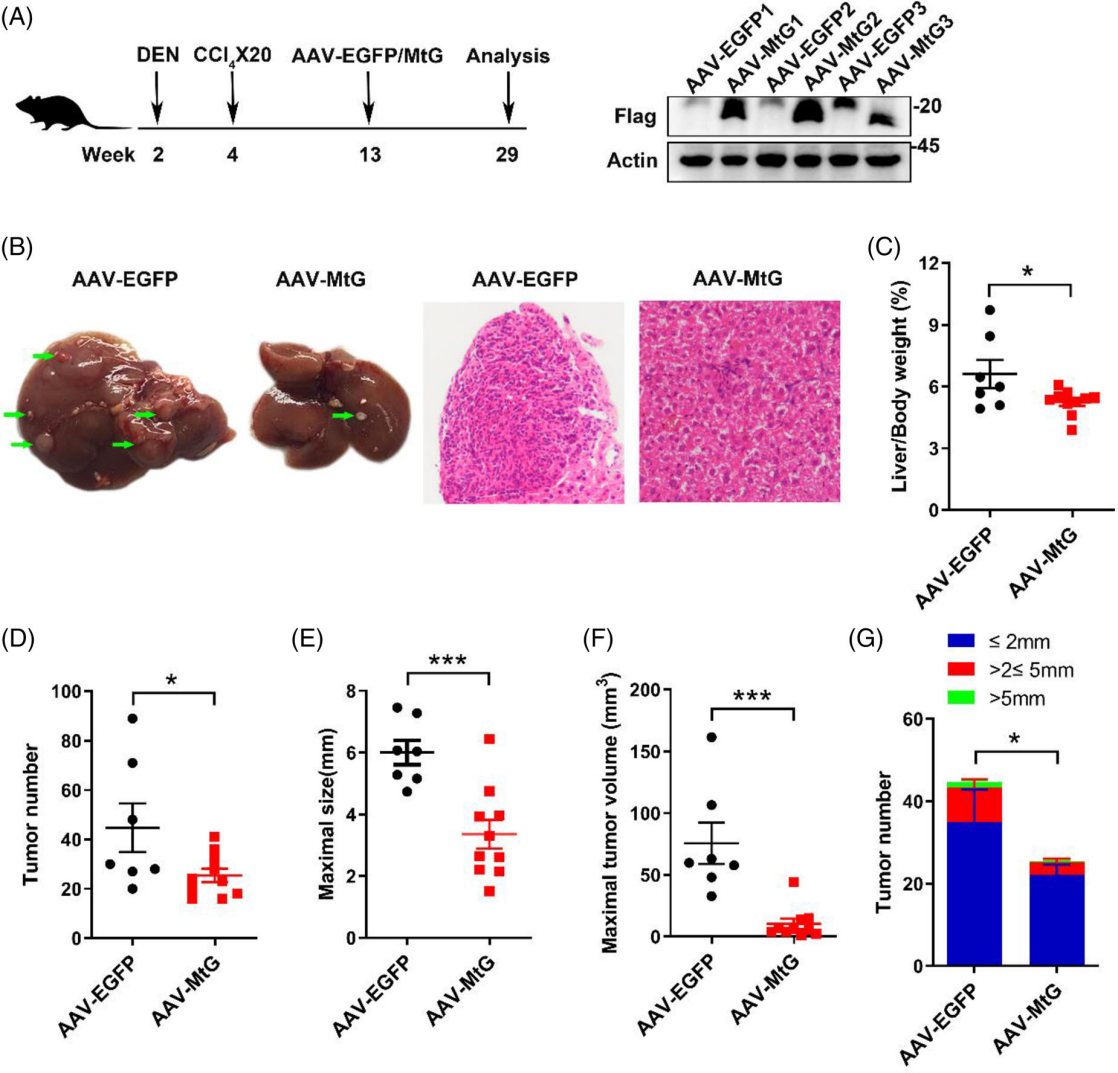
Mitochondrial general control of amino acid synthesis 5 like 1 (GCN5L1) expression protects against diethylnitrosamine (DEN)‐ and carbon tetrachloride (CCl_4_)‐promoted hepatocellular carcinoma (HCC) development. Two‐week‐old wild‐type male mice were i.p. injected with DEN (25 mg/kg body weight), followed by i.p. injected with CCl_4_ (5 ml/kg body weight) weekly 20 times. Mitochondrial‐restricted GCN5L1 (MtG) was delivered to livers by AAV injection through the tail vein at 13 weeks of age (n = 7 for AAV‐EGFP, n = 10 for MtG). Mice were euthanized at 29 weeks of age for HCC analysis. (A) Schematic of the experimental design. Mitochondrial GCN5L1 expression was confirmed by immunoblotting. (B) Representative images and hematoxylin and eosin (H&E) staining of mouse livers from the indicated groups. Arrows indicate tumours. (C) Ratio of liver weight to body weight. (D) Tumour number. (E) Maximal size of tumours. (F) Maximal volume of tumours. (G) Number of liver tumours of the indicated sizes. Values are expressed as the mean ± standard error of the mean, *P < .05, ***P < .001 versus respective control groups by two‐tailed unpaired Student's *t* test or Bonferroni's multiple comparisons test

Given that GCN5L1 possesses distinct functions in mitochondria and lysosomes,^25^ we next determined whether mitochondrial‐restricted GCN5L1 (MtG) overexpression sufficed to inhibit liver tumour development. Since 80% of HCCs develop in fibrotic livers due to chronic liver injury, we introduced a mouse model combining DEN and CCl_4_, which promotes liver fibrosis. This model incorporates hepatic chronic injury and fibrogenesis to accelerate DEN‐induced HCC. Here, WT mice were injected with DEN at 2 weeks of age followed by CCl_4_ at 4 weeks of age. At 13 weeks of age, when mice had evidence of microtumours (approximately 1 mm diameter), MtG or control enhanced green fluorescent protein (EGFP) AAV was transduced into the mice via tail vein injections. Mouse livers were harvested at 29 weeks of age. EGFP and MtG expression was confirmed by immunoblotting of liver tissues (Figure 2A). We found that the mice with MtG overexpression had markedly smaller tumours (Figure 2B). The liver/body weight ratio and tumour number were significantly reduced in AAV‐MtG‐injected mouse livers compared with EGFP controls (Figure 2C,D). Moreover, maximal tumour size and volume were dramatically decreased in MtG‐overexpressing mouse livers (Figure 2E,F). The distribution assessment indicated that the number of various tumour sizes, especially the large tumours, was significantly decreased in MtG‐expressing mice (Figure 2G). The results indicated that mitochondrial‐localised GCN5L1 suppressed hepatic tumour cell growth.

### 3.2 GCN5L1 expression is reduced in DEN‐ and CCl_4_‐induced HCC

The levels of GCN5L1 and mitochondrial protein acetylation are significantly decreased during mouse liver regeneration.^21^ To examine the expression levels of GCN5L1 in HCC, we analysed HCC tumours and adjacent liver tissues from the combined DEN and CCl_4_ model (Figure S1A). We performed immunoblot analyses of tumours and their adjacent normal liver tissues and found that GCN5L1 protein levels were decreased in DEN‐induced HCC tumours in comparison with adjacent liver tissues (Figure S1B). Consistent with this result, GCN5L1 protein levels in the hepa1‐6 and hepa1c1c7 murine HCC cell lines were lower than those in primary hepatocytes (Figure S1C). Unexpectedly, adjacent liver tissue had more GLS1 expression than tumours, whereas the liver type glutaminase GLS2 was slightly increased in tumours compared with adjacent tissues (Figure S1D). At the same time, tumours had greater elevations in glutaminase activity than the adjacent liver tissues (Figure S1D). Taken together, these results indicated that GCN5L1 protein levels decreased in murine HCC cells and tumours, and the regulation of GLS1 and GLS2 needs to be addressed further during the development of HCC.

### 3.3 Deletion of GCN5L1 in HCC cell lines promotes cell proliferation via enhanced glutamine metabolism

**FIGURE 3:**
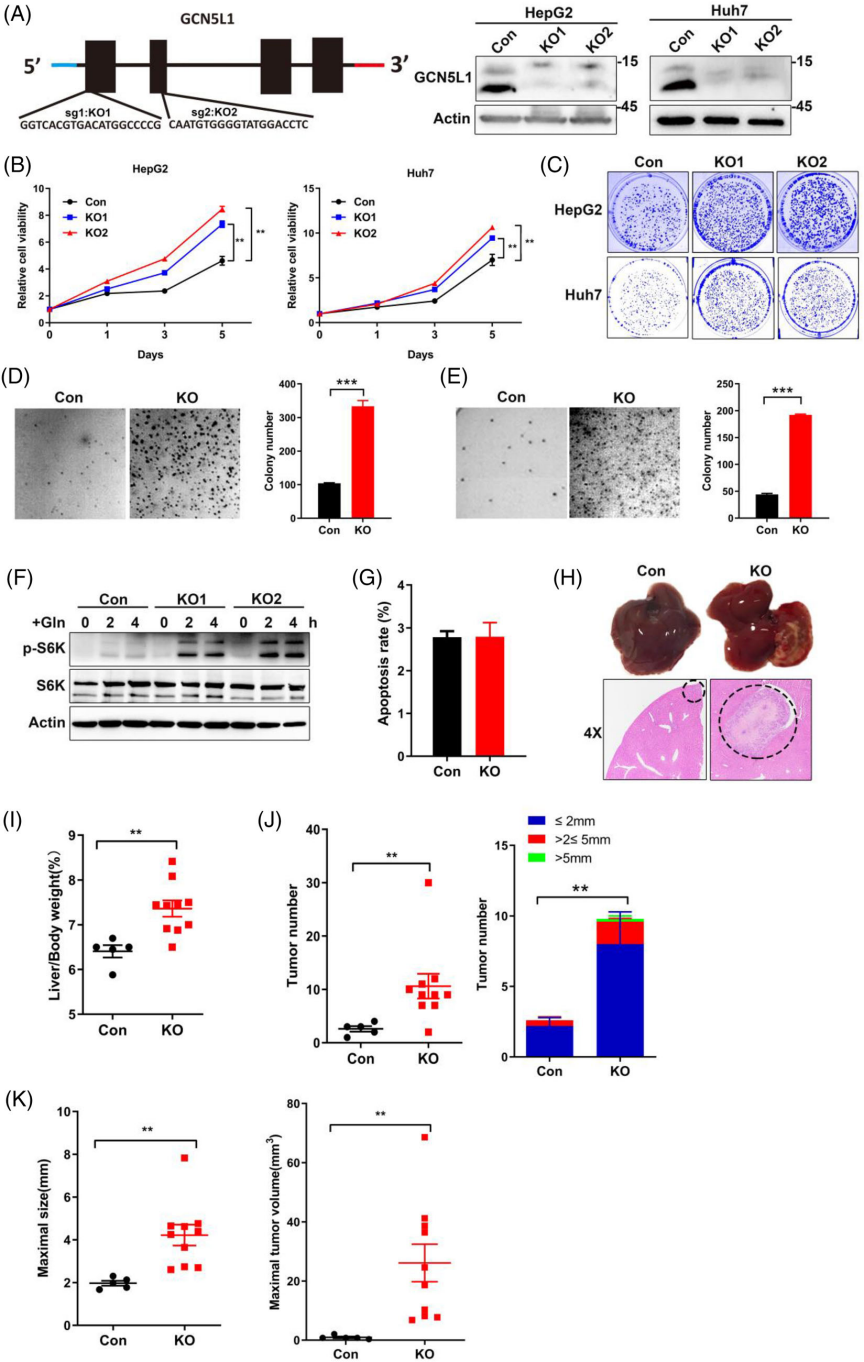
Deletion of general control of amino acid synthesis 5 like 1 (GCN5L1) in hepatocellular carcinoma (HCC) cell lines promotes cell proliferation and mammalian target of rapamycin complex 1 (mTORC1) activation. (A) GCN5L1 was depleted by the CRISPR system with small guide RNA (sgRNA) targeting exon 1 or exon 2. The knockout efficiency was confirmed by immunoblotting in the indicated cells. (B‐E) MTT assay (B), 2‐D colony formation assay (C) and representative soft agar images and colony numbers (D and E) of the indicated HepG2 or Huh7 cells with GCN5L1 depletion by sg2. Data are presented as the means ± standard deviation (SD) from three independent experiments (n = 3). **P < .01, ***P < .001. (F) GCN5L1‐deleted HepG2 or control cells were incubated in EBSS for 5 h and treated with 4 mM glutamine as indicated. Phosphorylation levels of S6K were analysed by immunoblotting. (G) Cell apoptosis assays were performed in HepG2 cells with GCN5L1 deletion and control cells. Data are shown as the means ± SD from three independent experiments. (H‐K) HepG2 cells (8 × 10^5^) with or without GCN5L1 depletion were injected into the left lobe of the liver of athymic nude mice (n = 5 for control, n = 10 for GCN5L1 KO). The mice were euthanized and examined for tumor growth 10 days after injection. The liver images and hematoxylin and eosin (H&E) staining are shown. Tumour regions are indicated by circles (H). Ratio of liver weight to body weight (I). The number of tumours and indicated tumour sizes are shown (J). Maximal tumour sizes and volumes are presented (K). Data are shown as the means ± standard error of the mean, **P < .01 versus respective control groups by two‐tailed unpaired Student's *t* test

The in vivo results support that GCN5L1 functions as an HCC tumour suppressor. To further investigate the mechanism whereby mitochondrial‐enriched GCN5L1 modulates HCC development, we employed CRISPR technology targeting exon 1 or exon 2 of GCN5L1 to delete endogenous GCN5L1 in human HCC cell lines (HepG2 and Huh7). Two small guide RNAs (sgRNAs) were evaluated, and their knockdown efficiency was confirmed by immunoblot analysis (Figure 3A). We then determined the consequence of GCN5L1 KO on HepG2 and Huh7 proliferation using MTT, 2D and 3D colony formation assays. We found that both sgRNAs similarly accelerated cell proliferation (MTT assay—Figure 3B) and by 2D and 3D Matrigel assays (Figure 3C‐E). Reintroduction of GCN5L1 or MtG restored cell proliferation in HepG2 cells with GCN5L1 deletion (Figure S2A‐C). Loss of GCN5L1 has been reported to activate mTORC1 by enhancing glutaminolysis during murine liver regeneration.^21^ Here, we tested whether ablation of GCN5L1 had the same metabolic profile in human HepG2 cells following GCN5L1 KO versus control cells cultured in Earle's Balanced Salt Solution (EBSS) with glutamine supplementation for 2 or 4 hours. Consistent with GCN5L1 LKO primary hepatocytes,^21^ mTORC1 signalling was highly activated by glutamine in GCN5L1 KO HepG2 cells (Figure 3F). Given that glutamine metabolism is critical to maintain GSH levels and cellular redox control,^26^ which regulates apoptosis, we analysed cell apoptosis in HepG2 cells following GCN5L1 depletion. We found that GCN5L1 KO did not affect apoptosis (Figure 3G). To determine the role of GCN5L1 deficiency in promoting tumour development, we injected GCN5L1 KO or control HepG2 cells into the livers of athymic nude mice. GCN5L1 depletion promoted tumour growth (Figure 3H) and increased the liver/body weight ratio (Figure 3I). We also found increased tumour number, maximal tumour size and volume (Figure 3J,K) in GCN5L1‐depleted xenografts. These results supported that GCN5L1 deletion promotes cell proliferation in vitro and in vivo and implied that the activation of mTORC1 in GCN5L1 deletion could promote this tumour cell growth.

### 3.4 Both GLS1 and GLS2 are involved in the regulation of cell proliferation in GCN5L1‐deleted HCC cells

Considering that metabolic regulation depends on enzyme activities rather than gene transcription, we focused on glutaminase activity and GLS1/GLS2 protein levels. It has been reported that GLS2, as a rate controlling enzyme in liver glutaminolysis, performs an inhibitory function in cell proliferation attributable to being a target of the p53 tumour suppressor with decreased expression in tumours.^27^ However, recent research has shown that the inhibitory function of GLS2 on proliferation is independent of its glutaminase activity.^15^, ^28^ Furthermore, GLS2 has been reported to be a critical glutaminase isozyme in luminal‐subtype breast cancer.^14^ Although HCC has been shown to be a glutamine‐addicted tumour,^11^ whether GLS2 contributes to this development is still unclear. On the other hand, posttranslational modification is critical to modulate enzyme activities and metabolic adaptation, epitomised where GLS1‐K311 succinylation is enhanced in human pancreatic ductal adenocarcinoma,^26^ supporting that GLS lysine modification is involved in carcinogenesis. In mouse livers, K279 of GLS2 is regulated by GCN5L1, and this lysine residue is conserved between humans and mice. Thus, we hypothesised that GCN5L1 regulates glutaminase acetylation and activity to activate mTORC1 and promote HCC growth.

**FIGURE 4:**
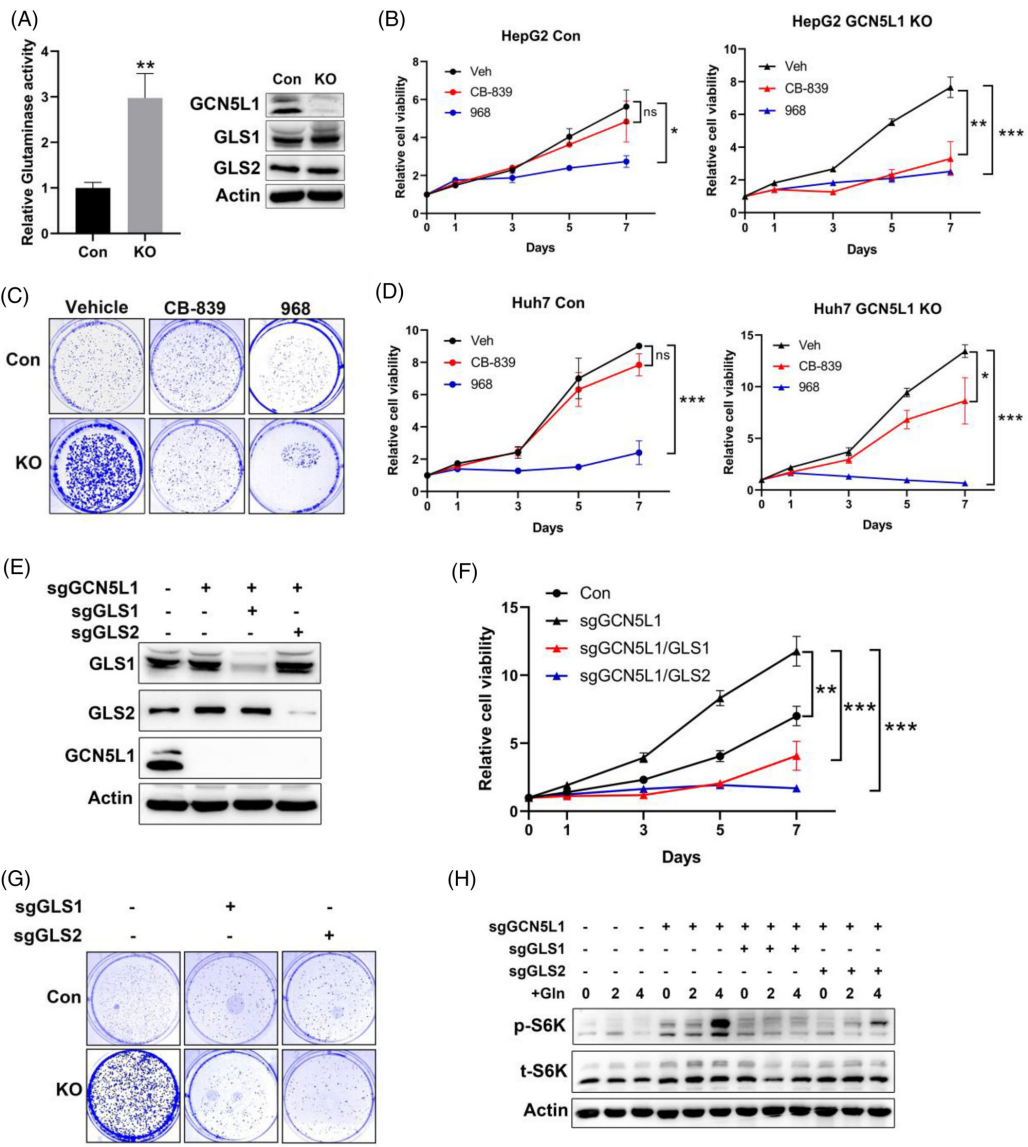
General control of amino acid synthesis 5 like 1 (GCN5L1) loss‐induced cell proliferation is dependent on the activity of glutaminase (GLS1 and GLS2) and mammalian target of rapamycin complex 1 (mTORC1). (A) Glutaminase activity was evaluated in HepG2 cells in the presence or absence of GCN5L1. Immunoblotting showed GLS1 and GLS2 expression levels in HepG2 cells. Data are presented as the means ± standard deviation (SD) from three independent experiments, **P < .01. (B and C) The GLS1 inhibitor CB‐839 (1 μM) and GLS2 inhibitor 968 (10 μM) were incubated with HepG2 cells with GCN5L1 deletion or control cells, and MTT assays (B) and 2‐D colony formation assays (C) were used to evaluate cell proliferation. (D) Huh7 proliferation was tested by MTT assay with/without CB‐839 (1 μM) and 968 (10 μM) in GCN5L1‐deleted cells and control cells. (E‐G) GLS1 or GLS2 was deleted by CRISPR in HepG2 cells with GCN5L1 deletion. Protein expression was confirmed by western blot (E). MTT assays (F) and 2‐D colony assays (G) were performed to evaluate cell proliferation. The cells were incubated in EBSS for 5 h, followed by incubation with 4 mM glutamine for the indicated times. p‐S6K levels were measured by immunoblotting (H). Data are presented as the means ± SD from three independent experiments

To explore the mechanism by which GCN5L1 levels alter HCC development and proliferation and the role of GLS1/GLS2 in HCC cell proliferation and glutamine addiction, we first measured glutaminase activity in GCN5L1‐deleted HepG2 cells. Here, GCN5L1 KO cells showed increased glutaminase activity without changes in GLS1 or GLS2 protein levels (Figure 4A). To confirm the role of GLS1 and GLS2 in the regulation of HCC proliferation, we introduced three inhibitors: CB‐839, bis‐2‐(5‐phenylacetamido‐1,3,4‐thiadiazol‐2‐yl)ethyl sulfide (BPTES) and Compound 968 (968). As reported, CB‐839 and BPTES are GLS1‐specific inhibitors, whereas 968 blocks both GLS1 and GLS2 activities and has a better inhibitory effect on GLS2 than GLS1.^14^ We then tested the inhibitory effects of these chemicals in 293T cells overexpressing GLS1 or GLS2. 293T cells were transfected with HA‐tagged GLS1 or Myc‐tagged GLS2, and 36 h after transfection, the cells were incubated with different doses of CB‐839 or 968 for 2 h. Then, glutaminase activities were measured to confirm the appropriate dose for blocking glutaminase activity. The lowest doses of CB‐839 at 1 μM and 968 at 10 μM performed adequate inhibitory effects on GLS1/GLS2 (Figure S3A,B). Meanwhile, we found that CB‐839 and BPTES inhibited GLS1 activity by over 80%, with a lesser effect against GLS2 activity (CB‐839 ∼42% reduction or BPTES ∼47% reduction) (Figure S3C,D). In contrast, 968 inhibited 19% of GLS1 activity but 88% of GLS2 activity (Figure S3C,D). These data indicated that CB‐839 and BPTES were specific GLS1 inhibitors and that 968 was more effective in inhibiting GLS2. We then used CB‐839 and 968 to test which glutaminase isozyme governed higher glutaminase activity in GCN5L1‐deleted HCC cells. MTT and colony formation assays showed that both CB‐839 and 968 blocked HepG2 growth following GCN5L1 ablation, implicating both GLS1 and GLS2 as important in GCN5L1 depletion‐mediated HepG2 cell proliferation (Figure 4B,C). However, 968 had a greater inhibitory effect than CB‐839 on control cell proliferation, suggesting that GLS2 rather than GLS1 was required for HepG2 cell glutamine addiction (Figure 4B,C). We observed a similar phenotype in Huh7 cells (another homosapien hepatocyte‐derived carcinoma cell line). Both CB‐839 and 968 blocked the enhanced cell proliferation in GCN5L1 deletion, and only 968 could significantly inhibit control Huh7 growth (Figure 4D). These data support a role for GLS2 in the control of HCC cell growth. However, both GLS1 and GLS2 contributed to cell proliferation following the ablation of GCN5L1. To confirm this result, we employed sgRNA depletion of GLS1 or GLS2 in GCN5L1‐deleted HepG2 cells (Figure 4E). Depletion of either GLS1 or GLS2 inhibited the excess growth evident in GCN5L1 KO cells (Figure 4F). Again, we used a colony formation assay to confirm this result (Figure 4G). GLS1 or GLS2 deletion blocked control HepG2 cell growth, which indicated the critical role of GLS1 and GLS2 in HCC cell proliferation (Figure S3E). We then evaluated mTORC1 activation in HepG2 cells with deletion of GLS1/GLS2. mTORC1 was activated in GCN5L1 KO cells and this activation was blocked by either deleting GLS1 or GLS2 (Figure 4H). Taken together, these results demonstrated that both GLS1 and GLS2 are critical to promote GCN5L1 deletion‐mediated facilitation of HCC cell proliferation.

### 3.5 Compound 968 inhibits DEN‐ and CCl_4_‐induced HCC development

**FIGURE 5:**
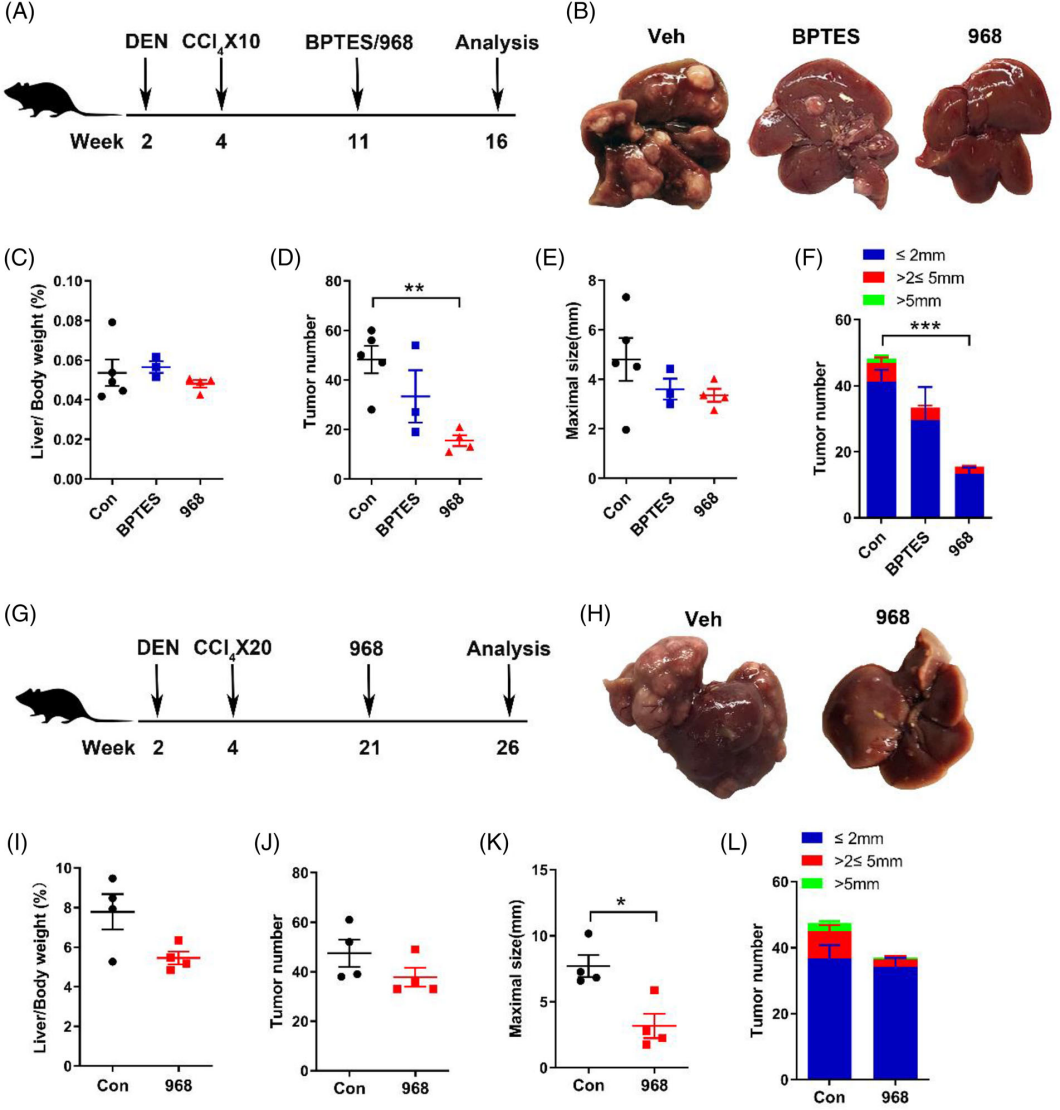
Glutaminase inhibitors protect against the early and late stages of hepatocellular carcinoma (HCC) development in diethylnitrosamine (DEN)‐treated mice. (A‐F) Wild‐type (WT) male mice were treated with DEN (25 mg/kg i.p. at the age of 2 weeks) and 10 carbon tetrachloride (CCl_4_) (5 ml/kg i.p.) injections and either saline (n = 5), bis‐2‐(5‐phenylacetamido‐1,3,4‐thiadiazol‐2‐yl)ethyl sulfide (BPTES) (n = 3) or 968 (n = 4), starting at 11 weeks of age until they were sacrificed. (A) Schematic of the experimental design. (B) Representative images of mouse livers from the indicated groups. (C) Ratio of liver weight to body weight. (D) Tumour number. (E) Maximal size of tumours. (F) Number of liver tumours of the indicated sizes. (G‐L) WT male mice were treated with DEN, 20 CCl_4_ injections and either saline (n = 4) or 968 (n = 4), starting at 21 weeks of age until they were sacrificed. (G) Schematic of the experimental design. (H) Representative images of mouse livers. (I) Ratio of liver weight to body weight. (J) Tumour number. (K) Maximal size of tumours. (L) Number of liver tumours of the indicated sizes. Values are presented as the mean ± standard error of the mean, *P < .05, **P < .01 by two‐tailed unpaired Student's *t* test or Tukey's multiple comparisons test

Given that GLS2 appears to play a larger role than GLS1 in glutamine consumption in HCC cell lines, we investigated whether GLS2 inhibition could have greater clinical efficacy in blunting HCC development. Here, we supplemented DEN‐ and CCl_4_‐induced HCC with either BPTES or 968. WT mice were administered DEN at 2 weeks of age, followed by weekly injection of CCl_4_ from 4 weeks of age for 10 weeks. At 11 weeks of age, these mice showed obvious albeit small (< 2 mm) tumours. The mice were then randomly separated into three groups to receive an additional i.p. injection of BETES, 968 or vehicle (Figure 5A,B). At 16 weeks of age, both BPTES‐ and 968‐treated mice had similar liver/body ratios compared to control mice (Figure 5C). However, 968 but not BPTES‐treated mice had significantly fewer tumours than control mice (Figure 5B,D). The maximal tumour sizes were slightly decreased in both BPTES‐ and 968‐treated mice (Figure 5E). The distribution assessment indicated that the number of various tumour sizes was significantly decreased in 968 treated mice (Figure 5F). Next, we tested whether compound 968 could be an effective therapeutic treatment for later‐stage disease. Here, DEN‐ and CCl_4_‐injected HCC mice were injected with 968 at 21 weeks of age instead of 11 weeks of age, and the mice were expected to have multiple larger tumours. At 26 weeks of age, the mice were sacrificed for analysis (Figure 5G,H). 968 slightly decreased the liver/body weight ratio (Figure 5I) without modifying the number of tumours (Figure 5J). However, maximal tumour sizes in 968 treated mice were significantly smaller compared to the control mice (Figure 5K). The distribution assessment showed that 968 diminished large tumours but not small ones (Figure 5L). These data support that the inhibition of GLS2 activity by 968 has therapeutic benefit and suggest that GLS2, rather than GLS1, is the dominant isoform contributing to murine HCC development.

### 3.6 Deletion of GCN5L1 enhances both GLS1 and GLS2 activities by altering acetylation

**FIGURE 6:**
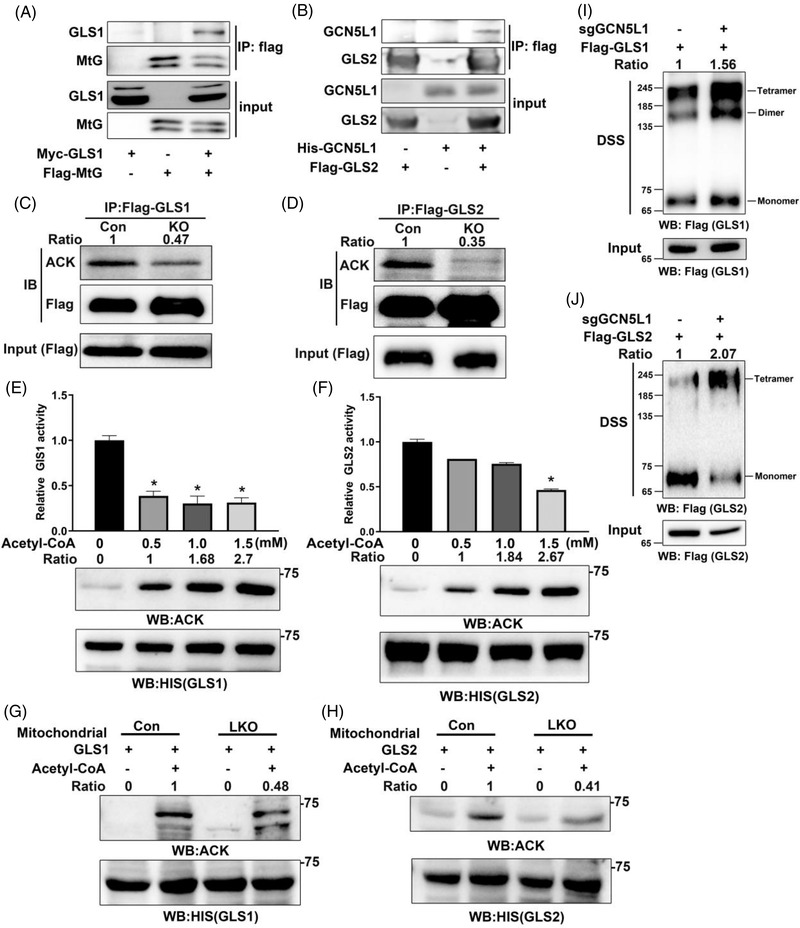
General control of amino acid synthesis 5 like 1 (GCN5L1) regulates the acetylation and oligomerization of glutaminase 1/2 (GLS1/GLS2). (A and B) GLS1 (A) or GLS2 (B) was transfected into 293T cells with GCN5L1. Cell lysates were immunoprecipitated with anti‐flag antibodies before immunoblotting analysis using anti‐Myc or anti‐His antibody as indicated. (C and D) Flag‐GLS1 or flag‐GLS2 was purified from HepG2 cells in the presence or absence of GCN5L1 by a pull‐down assay using a flag antibody. Immunoblot analyses were performed with the indicated antibodies. (E and F) Bacterially purified His‐GLS1 (E) and His‐GLS2 (F) were incubated with the indicated concentrations of acetyl‐CoA (0‐1.5 mM). The activities of these proteins were analysed using a glutaminase activity assay. Immunoblot analysis was performed with anti‐acetylated‐lysine antibody and anti‐His antibody. (G and H) Bacterially purified His‐GLS1 (G) and His‐GLS2 (H) were incubated with the liver mitochondrial protein of Con or GCN5L1 liver knockout (LKO) mice. Immunoblot analysis was performed with anti‐acetylated‐lysine antibody and anti‐His antibody. (I and J) Flag‐GLS1 or flag‐GLS2 was purified from HepG2 cells in the presence or absence of GCN5L1 by a pull‐down assay using a flag antibody and elution of 3 × flag peptides. Purified GLS1 or GLS2 was incubated with or without DSS. Immunoblot analyses were performed with flag antibody. The intensity of the blots was normalised to the input and is shown as fold change values versus Con in each lane. GLS1/2 oligomers normalised to monomers are shown versus control cells. One representative experiment of three performances is shown

Given that GLS1 and GLS2 protein levels were unchanged in GCN5L1 KO cells, we considered whether the functions of these proteins could be modulated by their posttranslational modifications, especially acetylation in mitochondria. To determine whether GLS1 and GLS2 are regulated by acetylation in GCN5L1‐deleted cells, we first confirmed that GCN5L1 interacted with GLS1 and GLS2 (Figure 6A,B). Next, we immunoprecipitated GLS1 or GLS2 from control and GCN5L1‐deleted HepG2 cells and evaluated their acetylation status by western blotting. Here, we found that the levels of both GLS1 and GLS2 protein acetylation were diminished in GCN5L1 KO HepG2 cells (Figure 6C,D). Furthermore, total acetylated proteins were accumulated by an anti‐acetyl‐lysine antibody from GCN5L1 KO and control HepG2 mitochondrial lysates, and the acetylation of GLS1/GLS2 was evaluated by immunoblotting GLS1/GLS2. We found that the acetylation levels of endogenous GLS1 and GLS2 were diminished in GCN5L1 KO HepG2 cells (Figure S4A,B). To determine whether GLS1 and GLS2 are acetylated in vitro, we incubated bacterially expressed recombinant His‐GLS1 or GLS2 with acetyl‐CoA and detected GLS1 or GLS2 acetylation, which was enhanced by increased concentrations of acetyl‐CoA within the tested range of 0–1.5 mM acetyl‐CoA (Figure 6E,F). Consistent with the glutaminase activity in GCN5L1‐deleted HepG2 cells (Figure 4A), an in vitro acetylation assay showed that inclusion of acetyl‐CoA significantly decreased the activity of bacterially purified GLS1 or GLS2 (Figure 6E,F). We also isolated liver mitochondrial proteins from control and GCN5L1 LKO mice, incubated them with purified GLS1 or GLS2 protein under 1.5 mM acetyl‐CoA and then detected acetylation. Consistent with previous cell experiments (Figure 6C,D), the acetylation of GLS1 or GLS2 significantly decreased after co‐incubation with the liver mitochondrial protein of GCN5L1 LKO mice (Figure 6G,H). To investigate GLS1/GLS2 acetylation in murine HCC tumours, we collected mitochondrial lysates from DEN plus CCl_4_‐induced tumours and adjacent tissues and the acetylation levels of both GLS1 and GLS2 were significantly downregulated in tumours compared with adjacent tissues (Figure S4C,D). Given that GLS1 or GLS2 activity is governed, in part, by enzyme oligomerisation and that acetylation or succinylation modifies this oligomerisation and therefore enzyme activity, we assayed GCN5L1 level‐dependent GLS1/2 oligomerisation. Flag‐tagged GLS1/GLS2 were extracted from HepG2 cells in the presence or absence of GCN5L1, and cross‐linked flag‐GLS1/GLS2 generated higher molecular weight species in the absence of GCN5L1 (Figure 6I,J). These data suggested that GCN5L1 regulated the acetylation of both GLS1 and GLS2 to modulate oligomer formation and therefore enzyme activity.

### 3.7 Glutaminase activity correlates with mTORC1 activation in HCC

**FIGURE 7:**
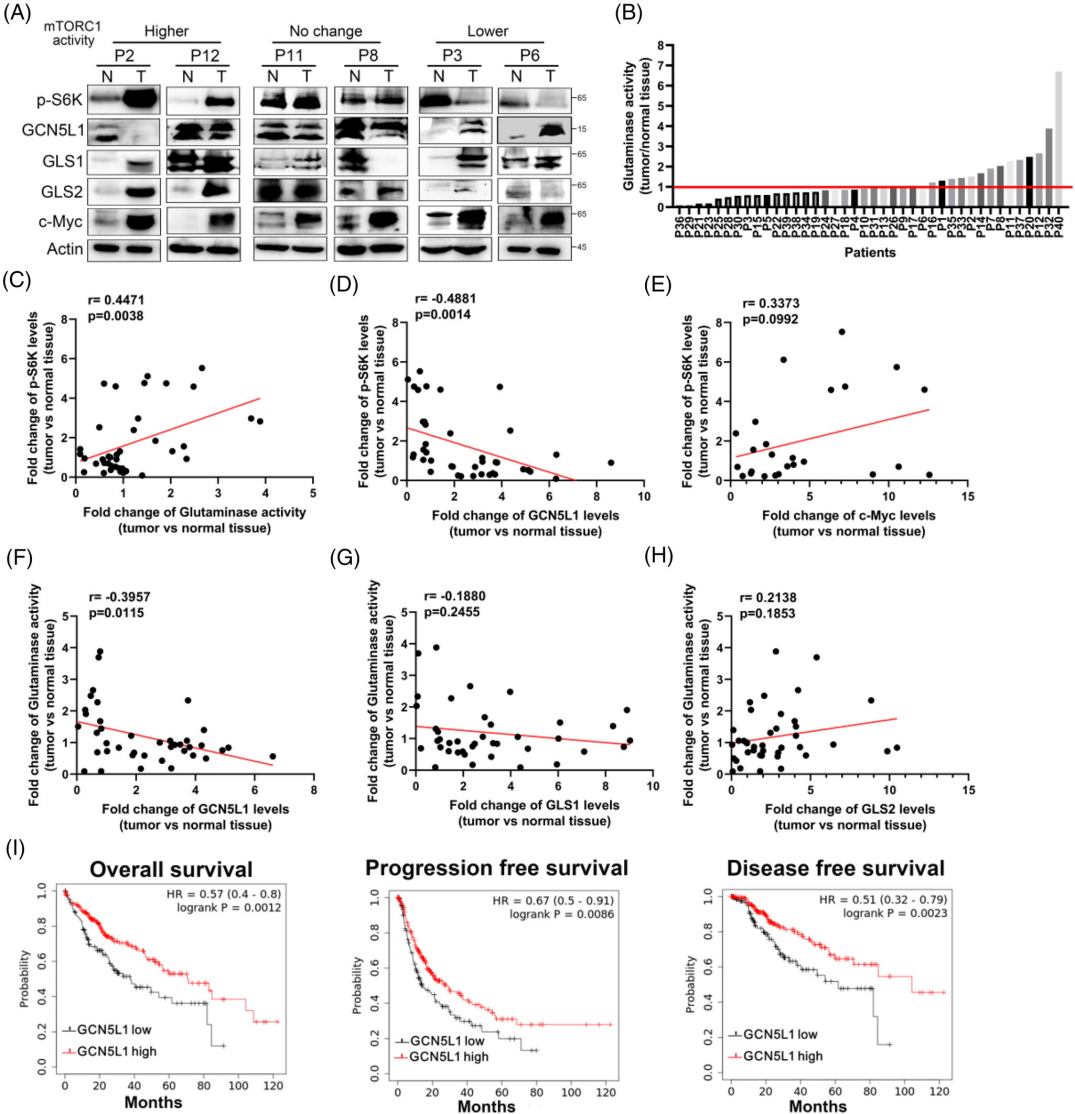
General control of amino acid synthesis 5 like 1 (GCN5L1) correlates with glutaminase activity and mammalian target of rapamycin complex 1 (mTORC1) activation in hepatocellular carcinoma (HCC) patients. Immunoblot analyses and glutaminase activity measurements of 40 human HCC specimens and their paired adjacent normal human liver tissues were performed. (A) Representative immunoblot images are shown according to mTORC1 activation status. (B) Comparative analysis of glutaminase activities between HCC specimens and their adjacent normal tissues was conducted. (C‐H) The fold changes in the protein levels of GCN5L1, p‐S6K, GLS1, GLS2 and c‐Myc in HCC tissues compared with adjacent tissues were analysed. The correlations between p‐S6K levels and glutaminase activities (C), between p‐S6K levels and GCN5L1 (D), between p‐S6K levels and c‐Myc (E), between glutaminase activities and GCN5L1 (F), between glutaminase activities and GLS1 (G) and between glutaminase activities and GLS2 (H) were analysed. (I) Kaplan–Meier plots of the overall survival (OS), progression‐free survival (PFS) and disease‐free survival (DSS) for HCC patients by GCN5L1 mRNA expression levels. All *P* values were calculated using the log‐rank test

In light of these findings, we hypothesised that glutaminase activity rather than GLS1/GLS2 expression is pivotal for mTORC1 activation in HCC development. Moreover, GCN5L1 acts as a tumour suppressor by regulating glutaminase acetylation and activity. To understand the clinical relevance of glutaminase activity, mTORC1 activation and GCN5L1 expression in human HCC, we examined glutaminase activities and the expression of p‐S6K, which indicates mTORC1 activation, and GCN5L1 in matched HCC specimens and adjacent normal liver tissues. Intriguingly, the data were quite variable, with only 14 out of 40 HCC specimens exhibiting lower GCN5L1 expression compared with adjacent tissues. Consistent with this variability of tumour GCN5L1 levels, we detected higher p‐S6K levels (over 1.5‐fold change) in 14 out of the total 40 HCC specimens compared to their adjacent non‐tumour tissues; 14 HCC specimens showed very modestly increased activity, and 12 HCC tumours had significantly lower (over 30% reduction) mTORC1 activities. Respective immunoblot images show these three patterns (Figure 7A). In parallel, glutaminase activity assessment similarly revealed variable glutaminase activity in HCC specimens in comparison with adjacent tissues (Figure 7B). We then analysed the correlation of tumour glutaminase activity with the expression of GLS1, GLS2, GCN5L1, c‐Myc and two major glutamine transporters downstream of c‐Myc, which are known to activate mTORC1 in HCC specimens.^16^ The correlation analysis showed that mTORC1 activation was significantly correlated with enhanced glutaminase activity (Figure 7C) as well as lower GCN5L1 protein levels (Figure 7D) but not with c‐Myc expression (Figure 7E) or transcripts of SLC1A5 and SLC7A6 (Figure S5A,B) in HCC specimens. Moreover, tumours with greater elevations in glutaminase activity significantly correlated with decreased GCN5L1 protein levels (Figure 7F) but not with GLS1 or GLS2 protein levels (Figure 7G,H). These results support that glutaminase activity represents an important aspect linking glutamine addiction to the malignant development of human HCC through the mTORC1 pathway. Additionally, GCN5L1 plays an important modifier role by modulating the acetylation and activity of glutaminase to regulate HCC development. Our follow‐up Kaplan–Meier analysis of liver cancer indicated that GCN5L1 expression was positively correlated with OS, PFS and DSS. We found that patients with high GCN5L1 expression survived longer than those with low GCN5L1 expression. For GCN5L1‐high patients and GCN5L1‐low patients, the OS medians were 71 versus 37.8 months, the PFS medians were 27.6 versus 13.83 months and the DSS medians were 104.17 versus 61.73 months, respectively (Figure 7I). At the same time, these data suggested that GCN5L1 plays a pivotal role in the treatment response and clinical outcomes of HCC patients. Collectively, these data suggest that targeting glutaminase or mTORC1 activity could be a potential therapeutic treatment for HCC expressing lower levels of GCN5L1.

## 4 DISCUSSION

Glutamine addiction is thought to be a metabolic signature of HCC. C‐Myc, as a primary oncogene in HCC, has been reported to activate mTORC1 to promote cell growth by increasing the transcription of glutamine transporters with the subsequent uptake of glutamine.^16^ At the same time, glutaminase, as a rate‐limiting enzyme in glutaminolysis, is encoded by two genes forming a liver‐type glutaminase (known as GLS2 or LGA) and a kidney‐type glutaminase (known as GLS1 or KGA). GLS2 is expressed only in the periportal area of the postnatal liver, whereas GLS1 is highly expressed in the kidney, brain, intestine, foetal liver, lymphocytes and cancer cells. GLS2, the primary type of glutaminase in the liver, has been shown to be replaced by GLS1 during HCC development.^12^ Furthermore, GLS1 is considered to be the critical form of glutaminase contributing to glutamine addiction in HCC, since GLS1 is highly expressed in HCC and is a target of c‐Myc. On the other hand, GLS2, as a target of the tumour suppressor p53, has been described as a tumour suppressor in HCC.^15^, ^27^ The function of GLS2 independent of its glutaminase activity is supposed to inhibit cell proliferation.^14^ It remains unclear whether the glutaminase activity of GLS2 contributes to HCC development. Thus, a mouse model with modulation of GLS2 activity is an appropriate strategy to evaluate the activity of GLS2 in HCC development. Utilising a chemical carcinogenesis model in GCN5L1 hepatic deleted mouse, which has altered GLS2 acetylation and activity, this study reveals that persistent activation of GLS2 in the liver promotes chemically induced HCC development.

In the current study, we present evidence that the absence of GCN5L1 promoted hepatic glutaminolysis by increasing GLS1 and GLS2 activities and that this metabolic remodelling increased both mTORC1 activity and HCC cell proliferation in response to chemically induced HCC models. Furthermore, this regulation was mediated, in part, by the change in acetylation of both GLS1 and GLS2 to modulate their oligomerisation and activity. Mitochondrial‐enriched GCN5L1 suppresses HCC development in the DEN plus CCl_4_ model. Furthermore, we show that both GLS1 and GLS2 contribute to glutamine addiction in HCC development. Thus, chemicals targeting both GLS2 and GLS1 together could be a more effective strategy to reduce HCC burden. It is worth noting that GCN5L1 has lower expression in five murine tumours compared to the adjacent non‐tumour tissues; however, this regulation is not uniform given that only one‐third of clinical cases show a reduction in GCN5L1 levels. Concurrently, mTORC1 activation, which may contribute, in part, to c‐Myc–driven HCC, was also only observed in 14 out of 40 HCC specimens. Further correlation analyses revealed the critical role of glutaminase activity rather than either GLS1 or GLS2 expression in HCC development. Interestingly, these data correlated with mTORC1 activation but were independent of c‐Myc expression. To further support the role of enzymatic activity in substrate uptake, mTORC1 activity showed a significant positive correlation with glutaminase activity but not with c‐Myc–controlled glutamine transporters. Our report highlights the importance of glutaminase activity in HCC development, whose regulation depends on posttranslational modification rather than their transcription. This novel finding indicates the possibility of modifying glutaminase regulation as a therapeutic strategy in HCC treatment.

GLS1 and GLS2 are located in the mitochondrial matrix, where approximately two–thirds of proteins have acetyl modification on one or more lysine residues to modulate their structure, activity and/or stability.^29^, ^30^ Acetyl‐CoA is a key node in glucose and fatty acid metabolism that enters the TCA cycle and generates ATP and functions as the provider of acetyl groups for protein acetylation. Research works have shown the importance of mitochondrial protein acetylation in metabolic regulation regarding obesity, diabetes, cardiovascular diseases and cancers.^31^, ^32^, ^33^ Currently, it has been revealed that the major regulators of mitochondrial protein acetylation are acetyl‐CoA and sirtuin 3 (Sirt3), which are mitochondrial protein deacetylases. Meanwhile, the mitochondrial acetyl‐CoA content is connected to metabolic status, such as obesity and nonalcoholic fatty liver disease (NAFLD), which are both associated with the development of HCC.^23^, ^34^, ^35^ Thus, the acetylation or other post translational modifications (PTMs) of glutaminase enzymes may be attractive candidates for drug screening as a strategy to alleviate the burden of HCC.

Finally, we found that decreased GCN5L1 levels, rather than alterations in GLS1 or GLS2 expression, are associated with human HCC tumours and support that changes in glutaminase activity are important in the pathogenesis of this disease. The exact mechanism underlying the modulation of GCN5L1 levels to regulate glutaminase activity in human HCC remains to be determined. We also found high levels of GLS1 (26 out of 40 cases over 1.5‐fold change) and GLS2 (25 out of 40 cases over 1.5‐fold change) expression in association with human HCC tumours, suggesting that further investigations are warranted to delineate the regulation and function of the two genes based on their roles in glutamine metabolism, non‐enzymatic functions and redox maintenance. Given the essential importance of glutamine metabolism and mTROC1 activation in other glutamine‐addicted cancers, GCN5L1 may contribute to the malignant progression of other types of tumours as well. At the same time, in the Kaplan–Meier analysis of liver cancer, we found that HCC patients with high GCN5L1 expression survived longer than those with low expression. Collectively, the detection of GCN5L1 expression may be a useful biomarker for the use of glutaminase inhibitors for therapeutics not only against HCC but also targeting other glutamine‐addicted malignancies.

## AUTHOR CONTRIBUTIONS

This study was conceived by Lu Zhu and Lingdi Wang; Taotao Zhang, Lingdi Wang and Lu Zhu designed the study; Experiments were performed by Taotao Zhang, Yunlong Cui, Yanjin Wu, Jiahui Meng, Jiaqi Zhang, Chunyu Zhang, Chenxi Yang, Lu Chen, Xue Bai, Kai Zhang, Kaiyuan Wu and Lingdi Wang; Data were analysed and interpreted by Taotao Zhang, Yunlong Cui, Yanjin Wu, Jiahui Meng, Linmeng Han, Jiaqi Zhang, Chunyu Zhang, Lingdi Wang and Lu Zhu; Yunlong Cui, Lu Chen provided clinical materials; Michael N. Sack provided conceptual advice and revised the manuscript. The manuscript was written by Taotao Zhang, Lingdi Wang and Lu Zhu with input from all authors.

## CONFLICT OF INTEREST

The authors declare no competing financial interests.

## Supporting information



Supporting Information
