# Supplementary material for: Mitochondrial GCN5L1 regulates glutaminase acetylation and hepatocellular carcinoma
Source: Clin Transl Med. 2022 May 11;12(5):e852. doi: 10.1002/ctm2.852 (PMC9091986; doi:10.1002/ctm2.852)
Supplement: Supplementary file 1 — Supporting Information [file CTM2-12-e852-s001.docx]

**Mitochondrial GCN5L1 regulates glutaminase acetylation and hepatocellular carcinoma**

Taotao Zhang^1^, Yunlong Cui^2^, Yanjin Wu^1^, Jiahui Meng^1^, Linmeng Han^1^, Jiaqi Zhang^3^, Chunyu Zhang^1^, Chenxi Yang^1^, Lu Chen^2^, Xue Bai^4^, Kai Zhang^4^, Kaiyuan Wu^5^, Michael N. Sack^5^, Lingdi Wang^3^, Lu Zhu^1^

**Detailed Materials and Methods**

**Mouse studies.**

All animal protocols were in accordance with Institutional Guidelines and approved by the Animal Care and Use Committee of Tianjin Medical University. All animals were kept under specific pathogen free (SPF) and temperature-controlled environment with a 12-hour light/dark cycle and housed with free access to water and normal chow diet. C57BL/6J mice and BALB/c nude mice for orthotopic tumor xenografts were purchased from Beijing Vital River Laboratory Animal Technology Co., Ltd. GCN5L1 liver knockout mice (LKO) have been described previously^1^ and compared to flox/flox littermate controls.

The DEN-induced liver cancer model was established as described previously.^2^ In brief, 14-day-old GCN5L1 LKO mouse and the littermate controls were injected with 25mg/kg body weight of DEN. After 23 weeks on normal chow, the mice were sacrificed, and liver tissues were collected. HCC mouse model combining DEN and carbon tetrachloride (CCl_4_) was described previously.^3^ Briefly, wild-type mice at 2 weeks of age were injected with DEN (25 mg/kg i.p.), followed by 10 or 20 weekly injections of CCl_4_ (5 ml/kg i.p., 10% dissolved in olive oil) at 4 weeks of age. For in-vivo hepatic mitochondrial GCN5L1 (MtG) expression, MtG or EGFP was packaged into AAV-TBG and delivered into mice (1x10^11^ total genome copies) intravenously via lateral tail vein injection. AAV was adjusted to 100 μl sterile phosphate buffered saline (PBS), pH7.4 (Gibco) before the injection.

Evaluation of tumor number and size was determined as described by counting the number of visible tumors and measuring the size of the largest tumor with a vernier caliber. After sacrifice, livers were explanted, digitally photographed, and weighed to calculate the liver: body weight ratio. Liver tissues were collected for further analysis. Tumor volume was calculated with the equation V (in mm^3^) = 0.52× length× width^2^.

The orthotopic mouse model was established as described previously.^2^ Six-week-old athymic nude male mice were anaesthetized by isoflurane, and a small opening was made on the upper abdomen to expose the left lobe of liver. 8×10^5^ cells (in 50 μl of DMEM: Matrigel (Corning 356237) =1:1) were injected into the liver by 300-μl micro-syringe. Then the incision was quickly sewn up with medical suture and suture needle and mice were placed on an electric blanket until being awakened. After 10 days, mice were euthanized and livers were explanted, digitally photographed, and weighed to calculate the liver: body weight ratio. Liver tissues were collected for further analysis. Tumor volume was calculated with the equation V (in mm^3^) = 0.52× length× width^2^.

**Constructs and antibodies.**

Expression plasmids for human GLS1 and GLS2 were constructed by standard molecular biology techniques and cloned in pShuttle-3xFlag, pShuttle-Myc or pET28A vector. Mitochondrial-restricted GCN5L1 (MtG) was previously described.^1^ The lentiviral sgGCN5L1, sgGLS1 or sgGLS2 vectors were generated via ligation of hybridized oligos (Supplementary table 1) into lentiCRISPR-v1 vector linearized with BsmBI (NEB) using T4 DNA ligase (NEB). Rabbit anti GCN5L1 was a kind gift from Prof. Dr. Michael Sack (NHLBI, NIH, Bethesda US). Rabbit anti p-p70S6K (9234), p70S6K (9202), ACK (9441), VDAC (4661), Tubulin (2146) were purchased from Cell Signaling Technology. Mouse anti flag (F1804) was from Sigma. Rabbit anti c-Myc (10828-1-AP) and GLS1 (12855-1-AP) were purchased from Proteintech. Rabbit anti GLS2 (AP6650D) was purchased from Abcepta, and β-actin (AC026,) was purchased from ABclonal. HRP goat anti-rabbit (AS014) or HRP anti-mouse (AS003) were purchased from ABclonal.

**Cell culture.**

HepG2, Huh7, Hepa1-6, hepa1c1c7 and HEK293T cell lines were cultured in Dulbecco’s modified Eagle’s medium (DMEM) (Gibco 11965118) supplemented with 10% fetal bovine serum (Gibco [10091148](https://www.thermofisher.com/order/catalog/product/10091148)) and 1% penicillin-streptomycin. Cells were used for experiments within 10-20 passages from thawing. All cells were authenticated via short tandem repeat testing. Mycoplasma detection was routinely performed to ensure cells were not infected with mycoplasma by using GMyc-PCR Mycoplasma Test Kit (Yeason, Shanghai, China). For MTT assays, Cells were seeded in 96-well plates (5000 cells/well) in appropriate growth medium, the rest of the steps were performed as previously described.^4^ For colony formation assay, cells were seeded in triplicate in 6-well plates (2000 cells/well). For inhibitor treatments, GLS inhibitors were added the following day after seeding the cells and was renewed every day. For amino acid starvation, cells were first switched to culture in Earle’s balanced salt solution (EBSS) for 5 hours, followed by supplemented with 4mM glutamine (Sigma, G7513) as indicated.

**Mitochondria isolation.**

Mitochondria were isolated from cells or mouse livers by two-step centrifuge strategy. Briefly, cells or liver tissues were homogenized in mitochondrial isolation buffer (225 mM mannitol, 75 mM sucrose, 0.5% BSA, 0.5 mM EGTA, and 30 mM Tris–HCl pH 7.4), and followed by centrifuge at 800g, 4°C for 5min, and then the supernatant was centrifuged at 9000g, 4°C for 10min. The pellet was collected as mitochondrial fraction.

**Virus production and infection.**

293T packaging cell line was used for lentivirus and AAV production. Briefly, for lentivirus packaging, post-transfection with Liposomal Transfection Reagent (Yeasen, Shanghai, China) according to the manufacturer’s instructions, viruses were collected twice after 48 and 72 hours. After passing through 0.45μm filters, virus was concentrated by PEG8000 (Solabio) and resuspended in DMEM. Appropriate amount of viruses was used to infect target cells in the presence of 8μg/ml polybrene (Solarbio). After lentivirus infection, cells were maintained in cultured medium with puromycin (2μg/ml). For AAV production, 293T cells were maintained in 150 mm plates. For each transfection, 10 μg of pAAV8 serotype packaging plasmid, 10 μg of pDF6 helper plasmid, and 10 μg of AAV2 plasmid carrying TBG promoter and the construct of MtG or EGFP were added to 1 ml of serum-free DMEM. 90 μl of PEI solution (1mg/ml) was then added to the mixture and incubated at room temperature for 10 min. The mixture was added to 20 ml of media and applied to each dish to replace the old growth media. Culture media were collected at 72h and 120h post transfection. The viral particles were then purified and titrated according to a previously published protocol.^5^

**Immunoprecipitation and western blotting.**

Proteins were extracted from cultured cells using cold RIPA lysis buffer (Yeasen) containing nicotinamide (Sigma), Trichostatin A (Sigma) and EDTA-free phosphatases/protease inhibitor cocktail (MCE), followed by immunoprecipitation and immunoblotting with the corresponding antibodies. Proteins were separated by 10% or 15% SDS gel, then transferred to PVDF membrane (Merck-ISEQ00010) and probed with the indicated antibodies including: GCN5L1(1:1000), p-p70S6K (1:1000), p70S6K (1:1000), ACK (1:1000), VDAC (1:1000), Tubulin (1:2000), c-Myc (1:1000), GLS1 (1:1000), GLS2(1:1000) and β-actin (1:50000). All primary antibodies were diluted in blocking solution (3% BSA, 0.1% Tween in PBS). The immunoblots were blocked for two hours at room temperature in 5% milk (PBS, 0.1%Tween) followed by an overnight incubation at 4°C in their respective diluted primary antibody solutions. Immunoblots were visualized using a chemiluminescence imaging system (MINICHEMI).

**Cross-linking analysis of glutaminase oligomerization.**

5 μg of flag-GLS1 or flag-GLS2 plasmid was transfected into GCN5L1 KO or control HepG2 cells, 36h after transfection, flag-GLS1 or flag-GLS2 was purified by anti-flag antibody and eluted by 3×flag peptide (150 μg /ml). 10 μl of eluted samples was run on SDS gels as loading control. 1 mM DSS (Sigma) was added to the purified flag-GLS1 or flag-GLS2 for 30min at RT. Cross-linked samples were run on SDS gels and then anti-flag antibody was used for immunoblotting analysis.

**Quantitative Real-Time PCR.**

Total RNA from cells or tissues were extracted with TRIzol RNA isolation reagents (Invitrogen) and reversely transcribed with a TransScript® II First-Strand cDNA Synthesis SuperMix (Yeasen) before quantitative RT-PCR analysis. RT-PCR was performed with an ABI 7300 Detection System using the SYBR Green PCR Mix (Yeasen) with appropriate primers. The data were normalized to expression of the control gene encoding β-actin in each individual sample. The RT-PCR primers are shown in (Supplementary table 1).

**Histology of mouse liver tissue.**

Mouse liver lesions were fixed in 4% paraformaldehyde overnight at 4°C and embedded in paraffin by two board-certified pathologists (S.R. and F.D.) in accordance with the criteria by Frith et al. All paraffin-embedded liver tissues were stained with hematoxylin and eosin (H&E) for analysis of morphologic changes.

**Glutaminase activity assay.**

Glutaminase activity assays were previously described.^6^ Briefly, mitochondria from HepG2 cells or HCC samples were used for endogenous glutaminase activity assays. To test the efficiency of glutaminase inhibitors, a plasmid encoding human GLS1 or GLS2 was transfected into 293T cells, 2h before cell harvest, inhibitors were incubated with the cells. Mitochondria were isolated and lysed in lysis buffer (50 mM HEPES pH7.9, 100 mM NaCl and 0.05% Triton X-100). 50 μg of mitochondrial protein was incubated in 50 mM Tris-acetate (pH 8.6), 0.25 mM EDTA and 20 mM glutamine for a volume of 90 μl at 37^o^C for 1h and was stopped by adding 10 μl of ice-cold 2.4M hydrogen chloride (HCl). The quenched reaction mixture was incubated with reaction mixture containing 160 mM Tris-HCl (pH 9.4), 400mM hydrazine, 5mM adenosine diphosphate (ADP), 2mM nicotinamide adenine dinucleotide (NAD), and 7.5U/ml glutamate dehydrogenase for a final volume of 220 μl. The reaction mixture was incubated at 37^o^C for 1h, and absorbance at 340nm was recorded for each sample against a water blank before (A1) and after final (A2) mixture incubation. The subtraction (A2-A1), which indicates NADH generation represented glutaminase activity for each sample. The residual protein samples were used for concentration analysis or immunoblotting against HA or Myc antibody as a loading control to calculate enzyme activity.

**Purification of recombinant proteins.**

His-GLS1, His-GLS2, and His-GCN5L1 were expressed in bacteria and purified. Briefly, corresponding constructs were expressed in the BL21 bacterial strain. The bacteria were cultured at 37°C until the OD reached 0.8. Protein expression was induced with 1mM IPTG overnight at 16°C. For His-tagged proteins, cleared lysates were loaded onto a Ni-NTA column, washed with 4 column volumes of 20 mM imidazole and subsequently eluted with 400 mM imidazole. Proteins were then desalted by washing with PBS using 10-kDa cut-through spin columns.

**In vitro acetylation assay.**

Bacterially purified proteins or soluble mitochondrial proteins (100μg) were incubated with different concentrations of acetyl-CoA (0-1.5mM) in TBS buffer (50 mM Tris-HCl, 150 mM NaCl adjusted to pH 8.0 at 37°C) with TSA and NAM at 37°C for 6h, and then subjected to SDS-PAGE and western blotting.

**Apoptosis analyses.**

Cells were stained with the Annexin V-EGFP apoptosis detection kit (Beyotime
Biotechnology), followed by cytometry (BD Biosciences). Annexin V-EGFP apoptosis detection kit that uses EGFP labeled recombinant human annexin V to detect phosphatidylserine on the surface of cell membrane during apoptosis. Briefly, 0.5-1 million HepG2 cells were collected and resuspended in 195μL Annexin V-EGFP binding solution, then 5μL Annexin V-EGFP and 10μL propidium iodide (PI) were supplied and incubated at room temperature for 20 min. Annexin V-EGFP and PI were analyzed by flow cytometry.

**Survival analysis.**

The effects of GCN5L1 gene on the survival of patients with liver cancer were performed using the Kaplan Meier Plotter online survival analysis tool (<https://kmplot.com/analysis/>). To obtain sufficient patient samples, the overall survival (OS), progression free survival (PFS) and disease-free survival (DSS) mode was used to conduct all the analyses.

**Statistical analysis.**

Data are presented as the mean ± s.e.m. unless otherwise indicated. Statistical analysis was conducted using unpaired two-tailed t-test, one-way or two-way analysis of variance (ANOVA), followed by Bonferroni’s post hoc tests with GraphPad Prism 9.0. Statistical significances are denoted as N.S. (not significant; P>0.05), *P < 0.05, **P < 0.01, ***P < 0.001. The numbers of experiments are noted in figure legends.

**Supplementary figures**

**
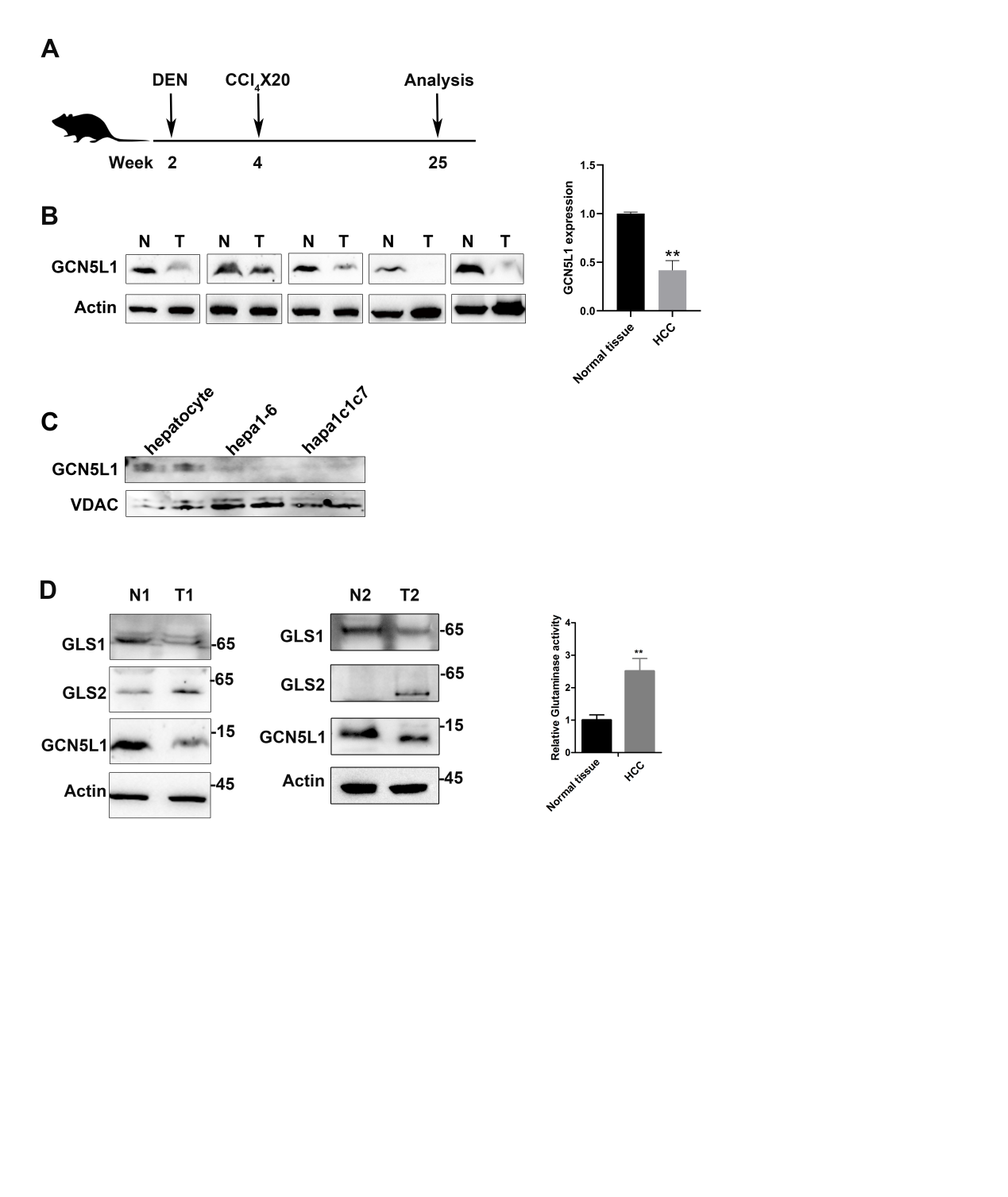
**

**Supplementary figure 1** GCN5L1 expression is decreased in DEN and CCl_4_-induced HCC. Two-week-old wildtype male mice were i.p. injected with DEN (25 mg/kg body weight) and followed by i.p. injected with CCl_4_ (5 ml/kg body weight) weekly for 20 times. Mice were euthanized at 25 weeks of age for HCC analysis. (A) Schematic of the experimental design. (B) Immunoblot analysis of GCN5L1 expression in HCC tumors and paired liver tissues. Averaged GCN5L1 levels were determined by densitometric quantification after normalization to that of adjacent normal tissues. n=5, Values are expressed as the mean ± s.e.m., **P<0.01 by two-tailed unpaired Student’s t-test. (C) Immunoblot analysis GCN5L1 expression in murine HCC cell lines and primary hepatocytes. (D) Representative image of immunoblot analysis of liver tumors (T) and adjacent normal tissues (N). The activities of mitochondrial were analyzed using a glutaminase activity assay. Averaged glutaminase activity were determined by densitometric quantification after normalization to that of adjacent normal tissues. n=5, Values are expressed as the mean ± s.e.m., **P<0.01 by two-tailed unpaired Student’s t-test.

**
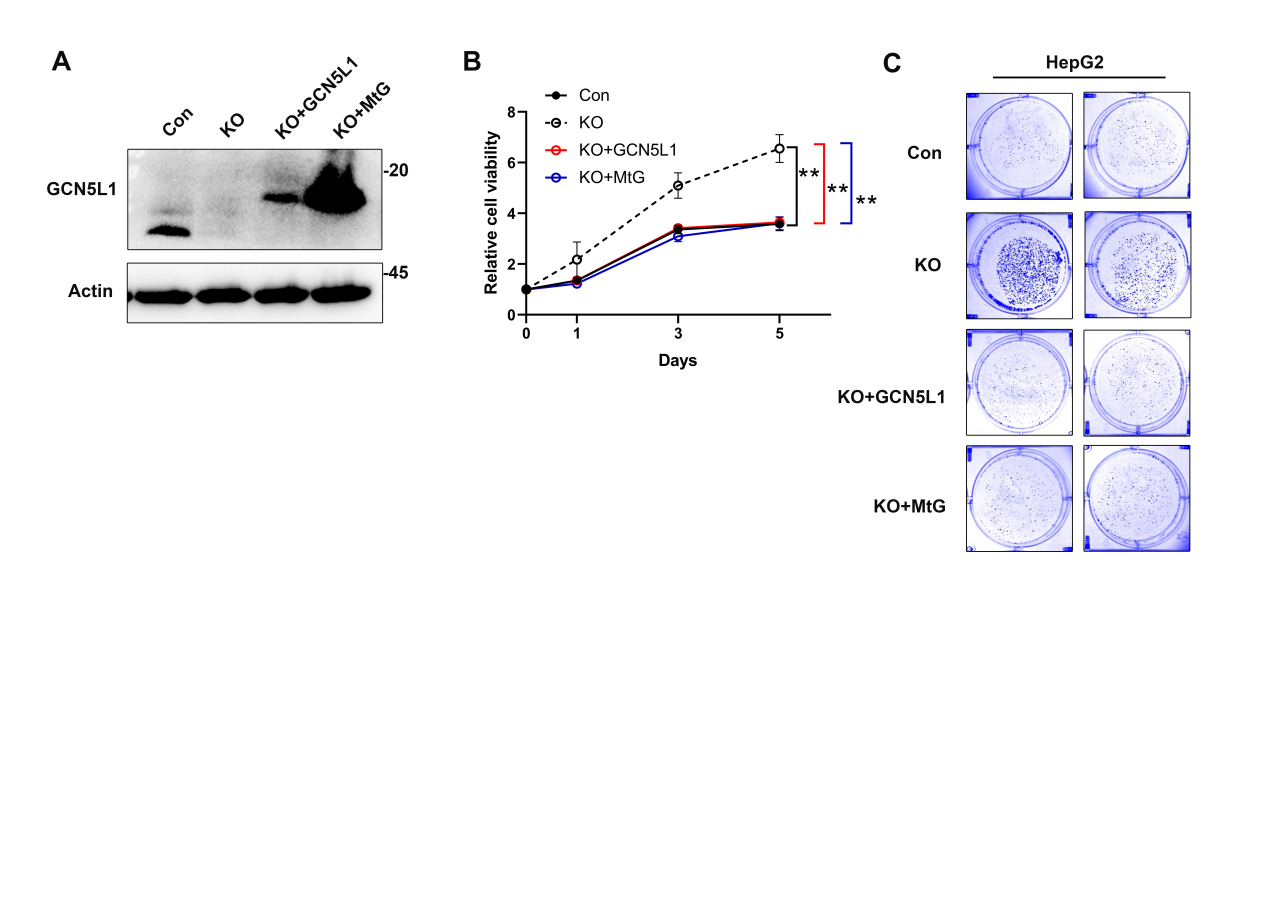
Supplementary figure 2** GCN5L1 or MtG overexpression in GCN5L1 deleted HepG2 cells reversed cell proliferation of GCN5L1 KO. (A) GCN5L1 or MtG was overexpressed in GCN5L1 KO cells and confirmed by immunoblotting. (B-C) MTT assay (B) and 2-D colony formation assay (C) of reintroduction of GCN5L1 or MtG in GCN5L1 deleted HepG2 cells. Data are presented as the means ± SD from three independent experiments (n=3), **p < 0.01, ***p < 0.001.

**
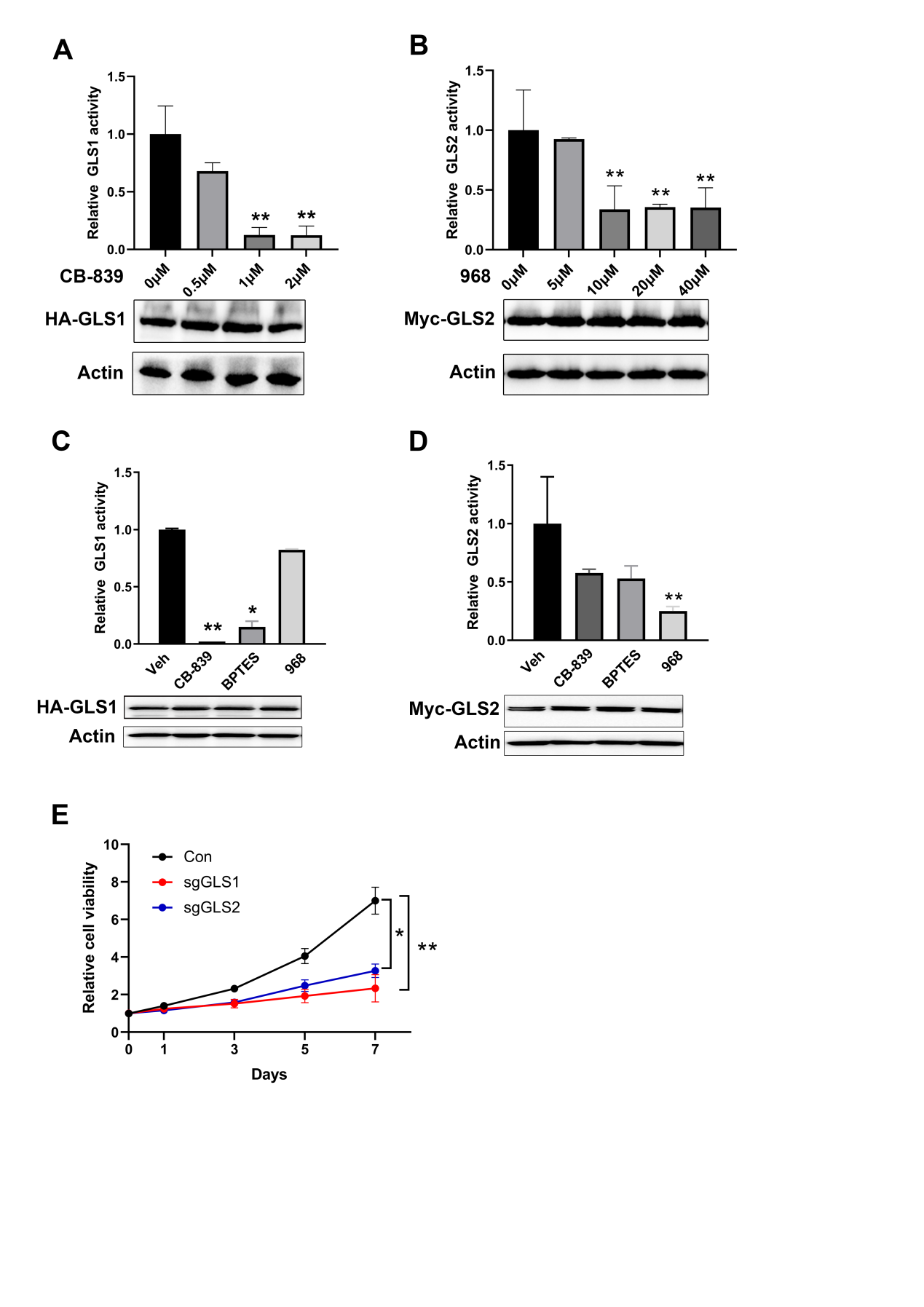
**

**Supplementary figure 3** The specific chemicals were analyzed to inhibit GLS1 or GLS2. 293T cells were transfected with HA-GLS1, Myc-GLS2 or empty vectors as background. 2h before cell harvest, cells were treated with chemicals and glutaminase activities were measured with subtraction of empty vector as background. Immunoblot confirmed the expression of GLS1 and GLS2. (A) and (C) Chemicals were tested to inhibit GLS1 activity. (B) and (D) Chemicals were tested to inhibit GLS2 activity. (E) MTT assay was done to evaluate HepG2 cell proliferation with GLS1/GLS2 deletion. Data are presented as the means ± SD from three independent experiments (n = 3), *P<0.05, **p < 0.01.

**
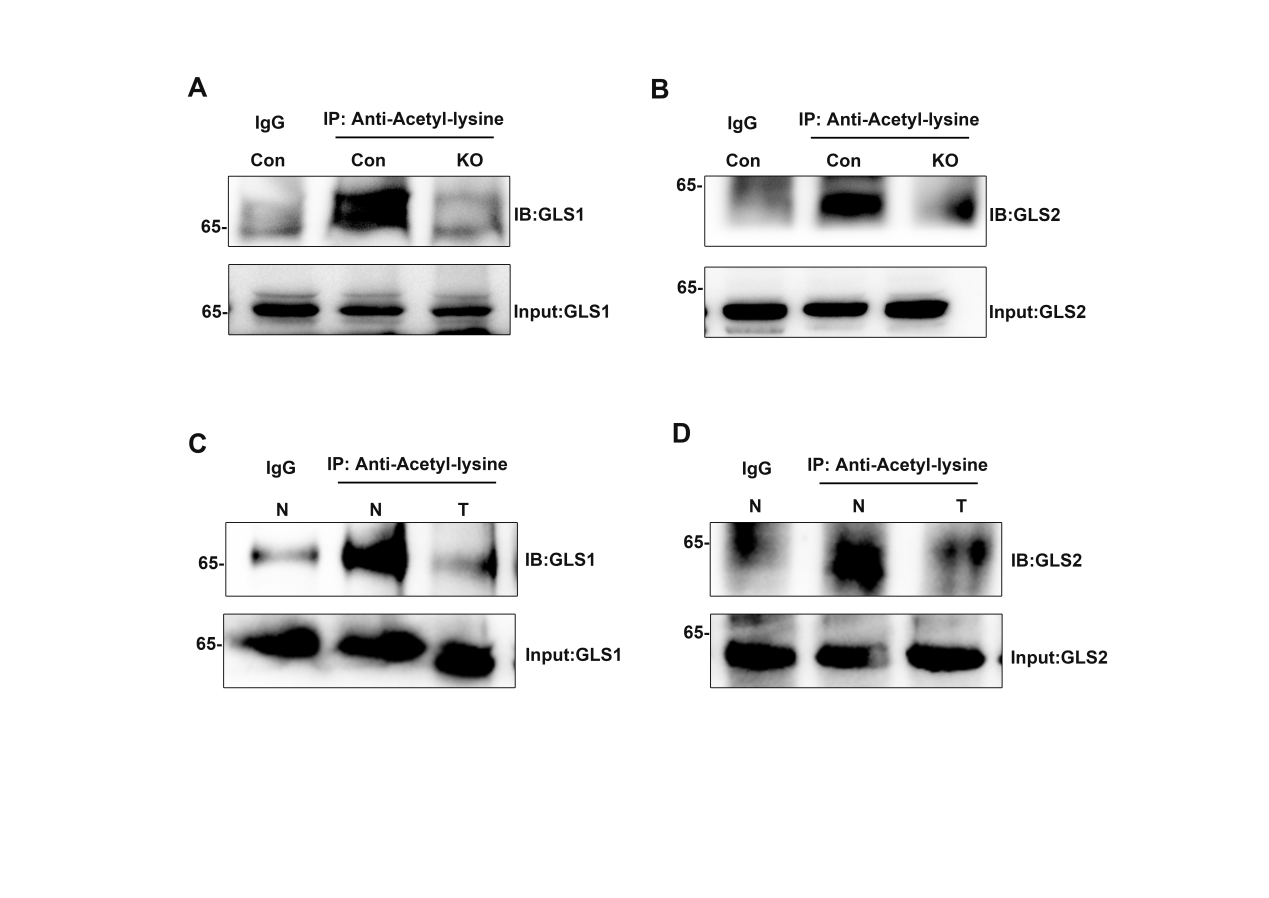
Supplementary figure 4** GLS1 and GLS2 acetylation were decreased in GCN5L1 KO cells and murine HCC tumors. Mitochondrial lysates from GCN5L1 KO and control HepG2 cells or murine HCC tumors (T) and adjacent liver tissues (N) were immunoprecipitated with anti-acetyl-lysine antibody and followed by analyzing GLS1/GLS2 acetylation with anti-GLS1 (A and C) or anti-GLS2 (B and D) antibodies. GLS1 or GLS2 expression was served as loading control. (A-B) Acetylation of endogenous GLS1/GLS2 in GCN5L1 KO and control HepG2 cells. (C-D) Acetylation of GLS1/GLS2 in murine HCC tumors and adjacent liver tissues.


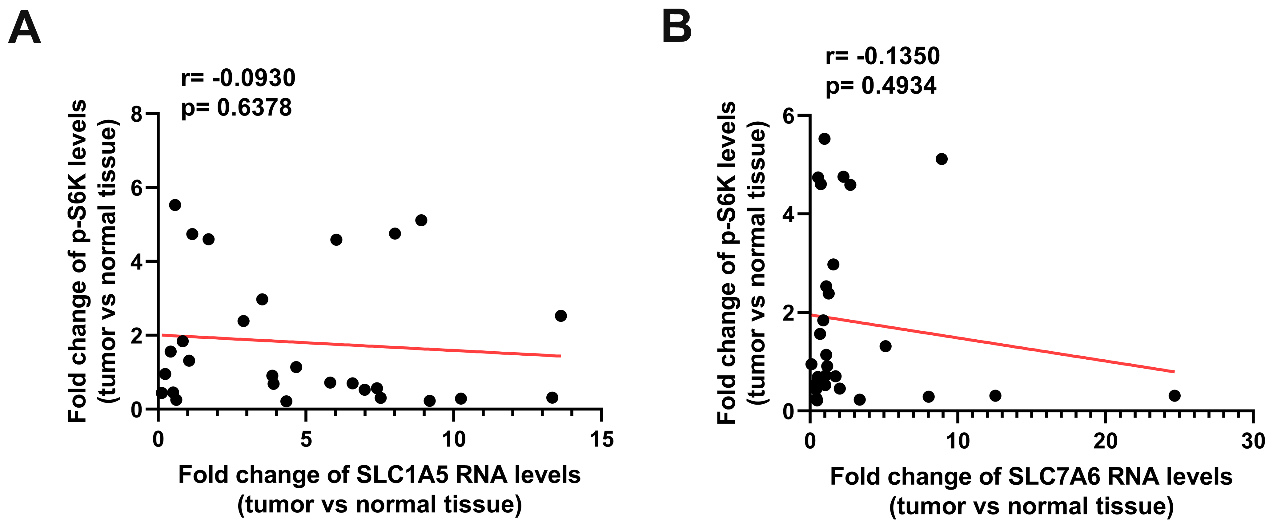


**Supplementary figure 5** Glutamine transporters were uncorrelated with mTORC1 activation in HCC patients (n=28 patients). The fold changes of RNA levels of SLC1a5 or SLC7a6 in HCC specimens compared with adjacent tissues were analyzed. The correlations between p-S6K levels and SLC1a5 (A) and between p-S6K levels and SLC7a6 (B) were analyzed.

**Supplementary table 1** Sequence of primers for generation of sgRNAs and quantitative real-time PCR

| Cloning primers | sequence (5'-3') |
| --- | --- |
| sgGCN5L1-1_F | CACCGGGTCACGTGACATGGCCCCG |
| sgGCN5L1-1_R | AAACCGGGGCCATGTCACGTGACC |
| sgGCN5L1-2_F | CACCGCAATGTGGGGTATGGACCTC |
| sgGCN5L1-2_R | AAACGAGGTCCATACCCCACATTG |
| sgGLS1_1F | CACCGGCGCGACTCCACCCGTGGTG |
| sgGLS1_1R | AAACCACCACGGGTGGAGTCGCGC |
| sgGLS1_2F | CACCGGAGCACGCATCCGCAGCCCG |
| sgGLS1_2R | AAACCGGGCTGCGGATGCGTGCTC |
| sgGLS2_1F | CACCGGCAGCCACCCTCGCCAACGG |
| sgGLS2_1R | AAACCCGTTGGCGAGGGTGGCTGC |
| sgGLS2_2F | CACCGTGCCATCGGCTATTATCTCA |
| sgGLS2_2R | AAACTGAGATAATAGCCGATGGCA |
| qPCR primers | **sequence (5'-3')** |
| Human GCN5L1_F | TGGACCATGAGGTGAAGACC |
| Human GCN5L1_R | TTCCTTGAGTGCCTGGTTGA |
| Human GLS1_F | AGGCACAGACATGGTTGGTA |
| Human GLS1_R | GGCAGAAACCACCATTAGCC |
| Human GLS2_F | CTCCATAAGCACCCTAGGCA |
| Human GLS2_R | ACAACAATGGCACCAGCATT |
| Human c-MYC_F | GCCAAGCTCGTCTCAGAGAAG |
| Human c-MYC_R | CAGAAGGTGATCCAGACTCTG |
| Huamn SLC1a5_F | CCTTTGGGACCTCTTCCAGT |
| Huamn SLC1a5_R | GATGAAACGGCTGATGTGCT |
| Huamn SLC7a6_F | TGGGACATGGGAAACCTCTC |
| Huamn SLC7a6_R | AGATGAGCGTCACAATTGGC |
| β-actin_F | CCCTGGAGAAGAGCTACGAG |
| β-actin_R | GGAAGGAAGGCTGGAAGAGT |
| 18S_F | GAGCCTGCGGCTTAATTTGA |
| 18S_R | AACTAAGAACGGCCATGCAC |

**References**

1 Wang, L. *et al.* GCN5L1 modulates cross-talk between mitochondria and cell signaling to regulate FoxO1 stability and gluconeogenesis. *Nat Commun* **8**, 523, doi:10.1038/s41467-017-00521-8 (2017).

2 Wu, Y. *et al.* Dual role for inositol-requiring enzyme 1alpha in promoting the development of hepatocellular carcinoma during diet-induced obesity in mice. *Hepatology* **68**, 533-546, doi:10.1002/hep.29871 (2018).

3 Dapito, D. H. *et al.* Promotion of hepatocellular carcinoma by the intestinal microbiota and TLR4. *Cancer Cell* **21**, 504-516, doi:10.1016/j.ccr.2012.02.007 (2012).

4 Liao, C. *et al.* Identification of BBOX1 as a Therapeutic Target in Triple-Negative Breast Cancer. *Cancer Discov* **10**, 1706-1721, doi:10.1158/2159-8290.CD-20-0288 (2020).

5 Challis, R. C. *et al.* Systemic AAV vectors for widespread and targeted gene delivery in rodents. *Nat Protoc* **14**, 379-414, doi:10.1038/s41596-018-0097-3 (2019).

6 Wang, L. *et al.* Mitochondrial General Control of Amino Acid Synthesis 5 Like 1 Regulates Glutaminolysis, Mammalian Target of Rapamycin Complex 1 Activity, and Murine Liver Regeneration. *Hepatology* **71**, 643-657, doi:10.1002/hep.30876 (2020).
